# POGZ Is Required for Silencing Mouse Embryonic β-like Hemoglobin and Human Fetal Hemoglobin Expression

**DOI:** 10.1016/j.celrep.2018.05.043

**Published:** 2018-06-12

**Authors:** Bjorg Gudmundsdottir, Kristbjorn O. Gudmundsson, Kimberly D. Klarmann, Satyendra K. Singh, Lei Sun, Shweta Singh, Yang Du, Vincenzo Coppola, Luke Stockwin, Nhu Nguyen, Lino Tessarollo, Leifur Thorsteinsson, Olafur E. Sigurjonsson, Sveinn Gudmundsson, Thorunn Rafnar, John F. Tisdale, Jonathan R. Keller

**Affiliations:** 1 Mouse Cancer Genetics Program, Center for Cancer Research, National Cancer Institute at Frederick, Bldg. 560/12-70, 1050 Boyles Street, Frederick, MD 21702, USA; 2 Basic Research Program, Leidos Biomedical Research Inc., Frederick National Laboratory for Cancer Research, Bldg. 560/32-31D, 1050 Boyles Street, Frederick, MD 21702, USA; 3 Department of Pediatrics, Uniformed Services University of the Health Sciences, 4301 Jones Bridge Road, Bethesda, MD 20814, USA; 4 Wexner Medical Center, Ohio State University, 460 West 12^th^Avenue, Columbus, OH 43210, USA; 5 Drug Mechanisms Group, Developmental Therapeutics Program, Leidos Biomedical Research, Inc., National Cancer Institute at Frederick, Frederick, MD 21702, USA; 6 The Blood Bank, Landspitali University Hospital, Snorrabraut 60, 105 Reykjavik, Iceland; 7 Iceland Genomics Corporation, Snorrabraut 60, 105 Reykjavik, Iceland; 8 Molecular and Clinical Hematology Branch, NHLBI/NIDDK, NIH, Bethesda, MD 20814, USA; 9 These authors contributed equally; 10 Present address: Molecular and Clinical Hematology Branch, NHLBI, NIH, Building 10, Room 9N112, Bethesda, MD 20814, USA; 11 Present address: Department of Pediatrics, Uniformed Services University of the Health Sciences, 4301 Jones Bridge Road, Bethesda, MD 20814, USA; 12 Lead Contact

## Abstract

Fetal globin genes are transcriptionally silenced during embryogenesis through hemoglobin switching. Strategies to derepress fetal globin expression in the adult could alleviate symptoms in sickle cell disease and β-thalassemia. We identified a zinc-finger protein, pogo transposable element with zinc-finger domain (POGZ), expressed in hematopoietic progenitor cells. Targeted deletion of *Pogz* in adult hematopoietic cells *in vivo* results in persistence of embryonic β-like globin expression without affecting erythroid development. POGZ binds to the *Bcl11a* promoter and erythroid-specific intragenic regulatory regions. Pogz^+/−^ mice show elevated embryonic β-like globin expression, suggesting that partial reduction of *Pogz* expression results in persistence of embryonic β-like globin expression. Knockdown of *POGZ* in primary human CD34^+^ progenitor cell-derived erythroblasts reduces *BCL11A* expression, a known repressor of embryonic β-like globin expression, and increases fetal hemoglobin expression. These findings are significant, since new therapeutic targets and strategies are needed to treat β-globin disorders.

## In Brief

Gudmundsdottir et al. show that POGZ represses embryonic globin gene expression in mouse and human erythroid cells, in part by regulating Bcl11a expression *in vitro* and *in vivo*. The molecular pathways regulated by POGZ may represent potential therapeutic targets to increase fetal globin expression in patients with sickle cell disease and β-thalassemia.

## Graphical Abstract

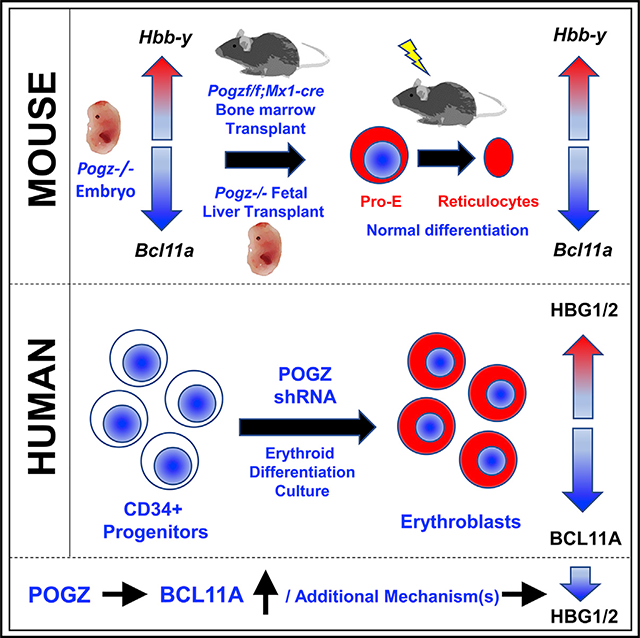

## INTRODUCTION

During mouse embryonic development, three distinct populations of erythroid cells are generated (Baron et al., 2013). The first are primitive erythroid cells, which arise from the yolk sac and mainly express embryonic β-like globins (*Hbb*-*bh1* and *Hbb*-*y*) and low levels of adult-type globins (*Hbb*-*b1* and *Hbb*-*b2*) (Kingsley et al., 2006; Palis, 2014). The second are definitive erythroid cells from the yolk sac that seed the fetal liver (FL). Initially, they express *Hbb*-*bh1* and *Hbb*-*y* and then switch to *Hbb*-*b1* and *Hbb*-*b2* expression (McGrath et al., 2011). The third population is hematopoietic stem cells (HSCs) that arise from intra-embryonic sites, including the aorta-gonad mesonephros region, that initially seed the FL and then home to the bone marrow and give rise to definitive erythroid cells. These erythroid cells express the adult *Hbb*-*b1* and *Hbb*-*b2* globins.

Sickle cell disease (SCD) and β-thalassemia are inherited human hemoglobin disorders, which result from globin gene mutations and represent a significant global health issue. Natural variations in fetal hemoglobin expression have been linked to the severity of disease outcome, such that individuals with higher fetal hemoglobin levels have less severe symptoms in SCD and β-thalassemia (Sankaran et al., 2010a). Genome-wide association studies identified three loci associated with increased fetal hemoglobin levels (Galarneau et al., 2010; Lettre et al., 2008; Uda et al., 2008), including *BCL11A*, which was subsequently shown to function as a transcriptional repressor of fetal hemoglobin (Sankaran et al., 2008). Conditional loss of *Bcl11a* in erythroid cells leads to increased embryonic β-like globin expression without affecting normal erythroid development, suggesting that BCL11A is a relevant therapeutic target (Xu et al., 2011). Recent experimental evidence confirmed that loss of *Bcl11a* expression in a preclinical model of SCD reversed sickling and end organ damage (Xu et al., 2011). Embryonic β-like globin is maintained in a repressed state by a multi-protein co-repressor complex, including BCL11A, GATA1, SOX6, and chromatin remodeling proteins, including Mi2β, HDAC1/2, LSD1/CoREST, and DMMT1 (Xu et al., 2010, 2013). These and other targets represent therapeutic opportunities to reactivate fetal globin to treat SCD and β-thalassemia (Bauer et al., 2012; Sankaran et al., 2008; Xu et al., 2013).

We identified a previously uncharacterized transcriptional regulator of hematopoiesis, POGZ (KIAA0461, ZNF280E), in a screen of a human hematopoietic progenitor cell line model (KG1) and its more differentiated progeny (Gudmundsson et al., 2007). POGZ is a zinc-finger containing protein, which binds to SP1, LEDGF, and heterochromatin proteins (Bartholomeeusen et al., 2009; Gunther et al., 2000; Nozawa et al., 2010), suggesting POGZ may have an important role in gene regulation; however, its function in hematopoiesis is currently unknown (Gudmundsson et al., 2007; Ishikawa et al., 1997; Nomura et al., 1994; Okazaki et al., 2003). Domain structure predictions by SMART analysis (Letunic et al., 2009; Schultz et al., 1998) indicate that POGZ has at least 8 C2H2 zinc fingers, suggesting it can bind DNA (Figure S1A). We show here that Pogz is expressed in normal mouse hematopoietic stem and progenitor cells (HSPCs), with the highest levels of expression in megakaryocyte erythroid progenitors (MEPs). We discovered that POGZ is essential for normal murine embryonic development, and uncovered a function of POGZ in the regulation of embryonic β-like globin expression *in vitro* and *in vivo*. Using mouse models that conditionally delete Pogz in adult mice, we demonstrate that *Pogz* is intrinsically required for normal globin switching, in part, by regulating *Bcl11a* expression. Furthermore, we show that knockdown of *POGZ* expression in human erythroid cells derepresses fetal globin expression. Our data provide evidence that Pogz is a regulator of mouse embryonic β-like globin expression and human fetal hemoglobin expression.

## RESULTS

### *Pogz* Is Expressed in Normal Murine Hematopoietic Stem and Progenitor Cells

**Figure 1.**
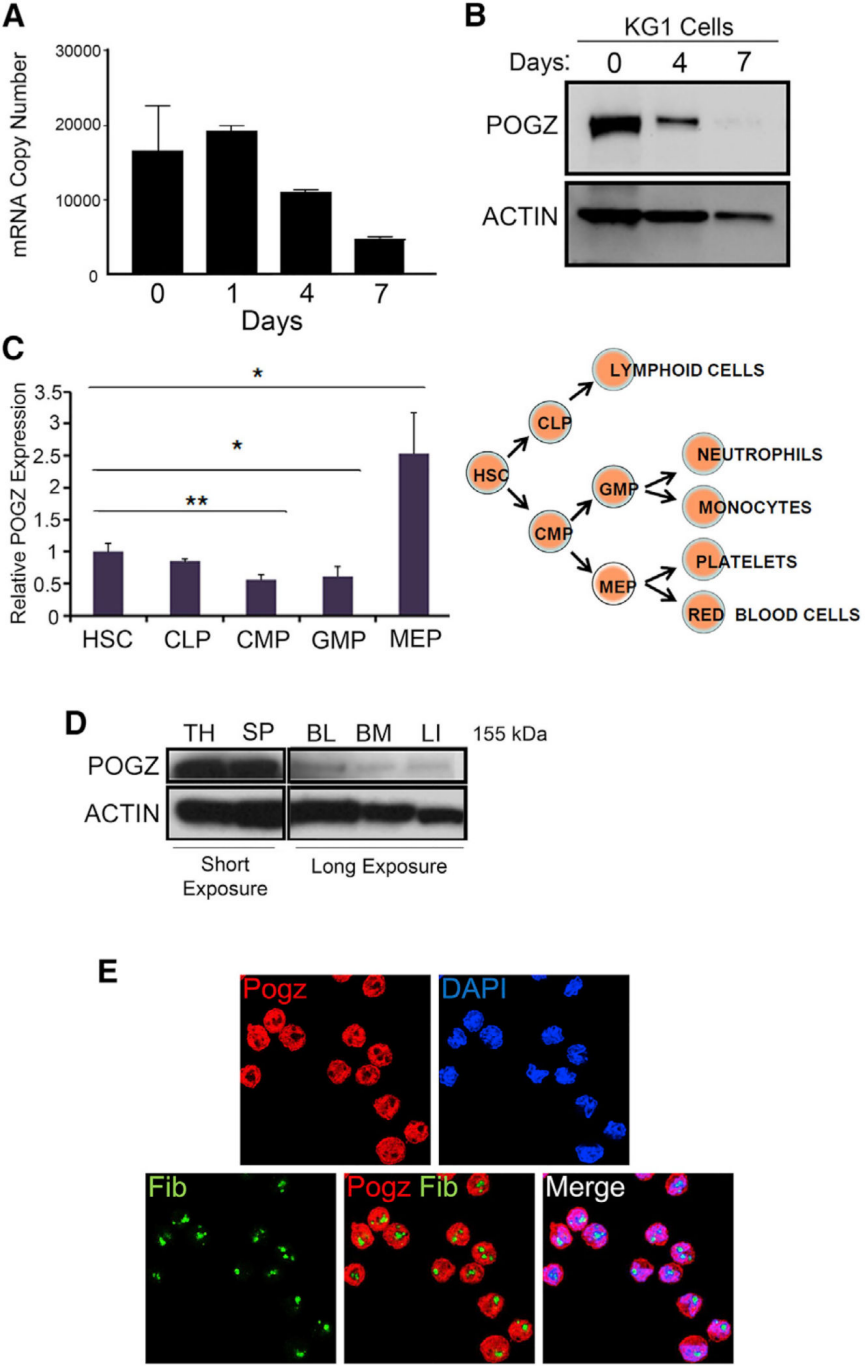
The Zinc-Finger Protein POGZ Is a Nuclear Protein Expressed in Human and Mouse Hematopoietic Cells (A) Real-time qPCR analysis reveals that *POGZ* expression is downregulated in KG1 cells after treatment with phorbol 12-myristate 13-acetate and tumor necrosis factor α (TNF-α). Experiments were performed in triplicate, and data are presented as mean ± SD (B) Western blot analysis shows reduced POGZ protein levels upon differentiation of KG1 cells. (C) Real-time qPCR analysis of *Pogz* expression in purified mouse hematopoietic stem cells (HSCs), common lymphoid progenitors (CLPs), common myeloid progenitors (CMPs), granulocyte macrophage progenitors (GMPs), and megakaryocyte erythroid progenitors (MEPs). Gene expression was normalized to β-actin expression. Experiments were performed in triplicate, and data are presented as mean ± SD. *p < 0.05; **p < 0.01. (D) Western blot analysis of POGZ in mouse tissue lysates indicates that POGZ is expressed in hematopoietic cells. (BL, blood; BM, bone marrow; LI, liver; SP, splenocytes; TH, thymocytes). Reduced exposure time is shown for thymus and spleen due to strong POGZ expression. (E) Immunofluorescence analysis of mouse erythroleukemia (MEL) cells. The cells were fixed, permeabilized, and stained with antibodies that recognize POGZ (red) and the nucleolar protein FIBRILLARIN (green). DNA was stained with DAPI (blue). POGZ is detected in the nucleus, but not the nucleolus.

We identified POGZ, a zinc-finger-containing protein, in a screen of the human hematopoietic progenitor cell line KG1, whose potential transcription factor activity and function in hematopoiesis were unknown (Gudmundsson et al., 2007). POGZ is expressed in KG1 cells, and POGZ RNA and protein levels are decreased during differentiation, suggesting that POGZ may function in hematopoietic cells (Figures 1A and 1B). POGZ is highly conserved from zebrafish to humans, with ~90% homology in coding sequence between human and mouse and 94% homology in amino acid sequence (Figure S1A). We performed a detailed analysis of *Pogz* expression in purified mouse HSPC populations and confirmed that *Pogz* is expressed in normal HSCs and common lymphoid progenitors (CLPs) and is reduced in common myeloid progenitors (CMPs) and granulocyte macrophage progenitors (GMPs), while *Pogz* expression is significantly higher in MEPs (Figure 1C). Inquiry of *Pogz* RNA expression in the BioGPS microarray database (Su et al., 2004) confirmed that *Pogz* is highly expressed in murine HSPCs and increased in MEPs and that *Pogz* is more broadly expressed in other tissues, including neural and eye tissue (Figure S1B). Finally, we compared POGZ protein expression in a limited tissue array and found that POGZ protein is highly expressed in adult mouse thymocytes and splenocytes, with lower levels of expression in peripheral blood cells (PBCs), bone marrow cells (BMCs), and liver cells (Figure 1D). Since POGZ is expressed in erythroid lineage cells, we used mouse erythroid leukemia (MEL) cells to examine the expression and subcellular localization of POGZ by immunofluorescence and determined that POGZ is mainly localized in the nucleus and is not present in the nucleolus (Figure 1E). Collectively, these results confirm that POGZ is expressed in normal mouse HSPCs, MEPs, and MEL cells, suggesting a potential role for POGZ in megakaryopoiesis and erythropoiesis.

### Reduced Output of Hematopoietic Cells and Deregulation of Genes Required for Erythropoiesis and Hemoglobin Switching in Pogz^−/−^ FL Cells

**Figure 2.**
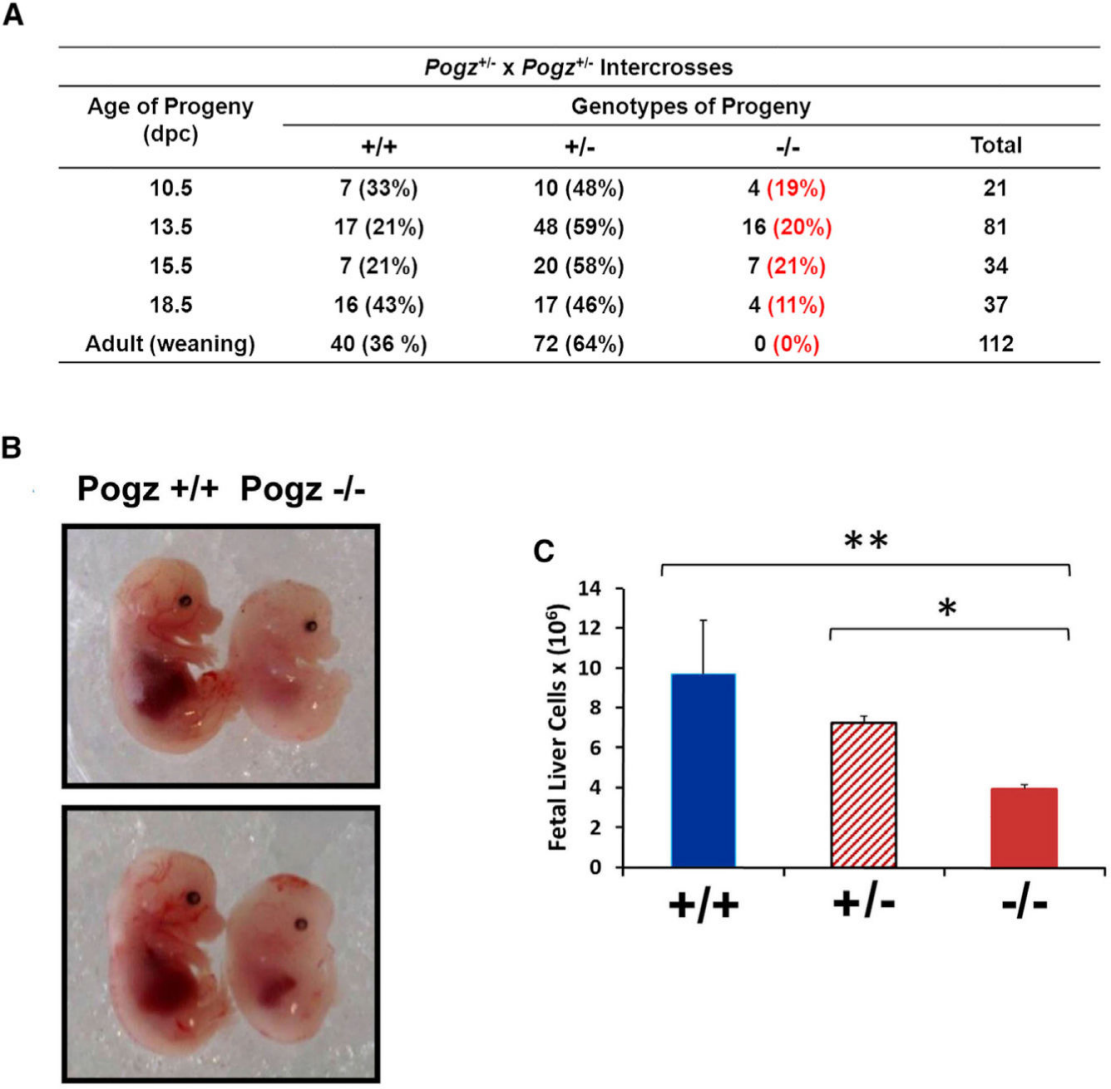
Pogz^−/−^ Embryos Are Runted, with Reduced Numbers of Fetal Liver Cells (A) Pogz^−/−^ mice do not survive beyond birth. Number and ratio of Pogz^+/+^, Pogz^+/−^ and Pogz^−/−^ progeny from intercrosses of mixed background (C57BL/6J × 1291/SVImJ mice) Pogz^+/−^ mice. The percentages within the parenthesis indicate observed progeny, and the expected outcome for these crosses is +/+ 25%, +/− 50%, and −/− 25%. (B) Gross morphology of E15.5 Pogz^+/+^ and Pogz^−/−^ embryos. (C) Total fetal liver cellularity in E16.5 Pogz^+/+^ (n = 2), Pogz^+/−^ (n = 2) and Pogz^−/−^ (n = 4) embryos. Data are presented as mean ± SD. **p < 0.01. These data are representative of 5 separate litters.

To uncover the physiological function of POGZ in hematopoietic development, we generated a mouse model to inactivate *Pogz* gene expression *in vivo* (Figures S1C–S1F) (Liu et al., 2003). We did not detect Pogz2^−/−^ pups at weaning in crosses of Pogz^+/−^ mice, suggesting that the Pogz^−/−^ mice died during embryonic development or shortly after birth. Further analysis showed that Pogz^−/−^ embryos rarely survived beyond embryonic day 16.5 (E16.5) when back-crossed 10 generations onto C57BL/6J background mice. Timed-pregnancy studies showed that some Pogz^−/−^ embryos were absorbed as early as E10.5, but we observed a consistent drop in animal survival around E15.5 (Figure 2A). The Pogz^−/−^ embryos were generally smaller and appeared anemic compared to their wild-type littermates; however, the precise cause of death is currently unknown (Figure 2B).

Since the FL is the major site of hematopoiesis in the embryo and Pogz^−/−^ embryos survive until E15.5–E16.5, we harvested FL cells from Pogz^+/+^ and Pogz^−/−^ embryos to examine lineage development by flow cytometry and performed differential gene expression analysis to identify potential pathways and target genes affected by the loss of *Pogz*. As expected, Pogz^−/−^ FL was significantly smaller, with fewer cells (Figure 2C). We found that the frequency of myeloid (macrophages [Mac1+Gr1−] and neutrophils [Mac1+Gr1+]) and B cells (CD19^+^) in Pogz^−/−^ FL was similar to Pogz^+/+^ FL; however, their total numbers were reduced due to the overall reduction in FL cellularity (Figures S2A–S2C). We observed increased frequencies of more primitive erythroid cells (S0–S2 cells) in Pogz^−/−^ FL (Figure S2D); however, the total number of the more mature erythroid cells was decreased at all stages of development (S3–S5) in Pogz^−/−^ FL (Figure S2E). Thus, erythroid, B, and myeloid cells are present in Pogz^−/−^ FL, but in greatly reduced numbers.

**Figure 3.**
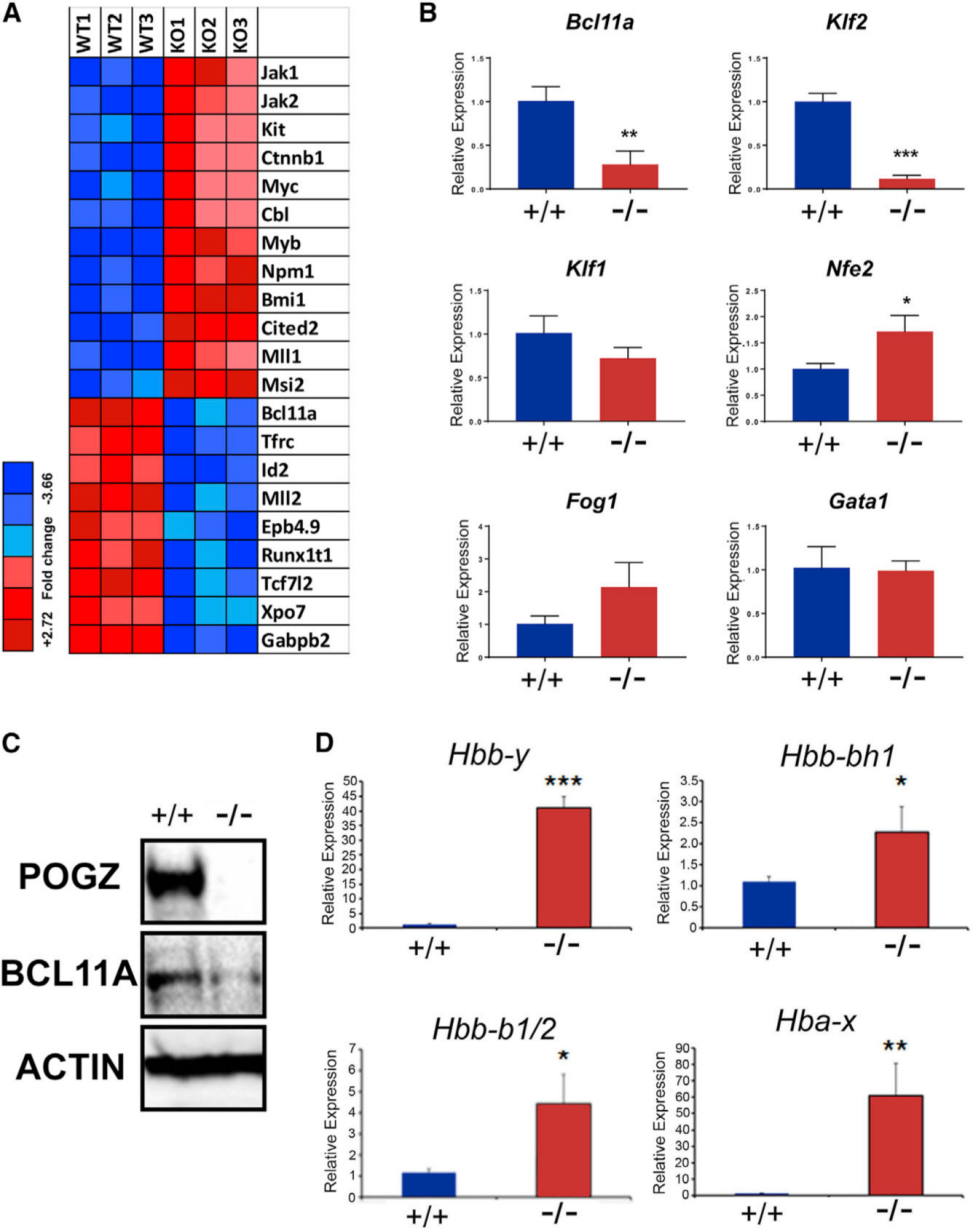
Decreased Expression of *Bcl11a* and Increased Expression of Embryonic β-like Globins in Pogz^−/−^ Fetal Liver Cells (A) Heatmap of differentially expressed genes in Pogz^+/+^ and Pogz2^−/−^ FL cells, indicating that genes expressed during erythroid lineage development are affected by loss of *Pogz* expression. RNA was isolated from E14.5 Pogz^+/+^ and Pogz^−/−^ FL cells (n = 3 for each genotype), and gene expression was analyzed on Affymetrix Mouse 430 2.0 oligonucleotide arrays (blue indicates low expression, and red indicates high expression). (B) *Bcl11a* and *Klf2* expression is downregulated in Pogz^−/−^ FL cells. RNA was purified from E16.5 Pogz^+/+^ and Pogz^−/−^ FLs, and *Bcl11a*, *Klf2*, *Nfe2*, *Klf1*, *Gata1*, and *Fog1* expression was analyzed by real-time qPCR. Gene expression was normalized to β-actin expression. Experiments were performed in triplicate, and data are presented as mean ± SD. *p < 0.05; **p < 0.01; ***p < 0.001. (C) Reduction in BCL11A protein levels in Pogz^−/−^ FL cells. Western blot analysis was performed on whole-cell lysates generated from E15.5 Pogz^+/+^ and Pogz^−/−^ FL cells. (D) Upregulation of embryonic β-like globins in Pogz^−/−^ FL cells. RNA was purified from E16.5 Pogz^+/+^ and Pogz^−/−^ FLs and *Hbb*-*y*, *Hbb*-*bh1*, *Hba*-*x*, and *Hbb*-*b1*/*2* expression analyzed by real-time qPCR. Gene expression was normalized to β-actin expression. Experiments were performed in triplicate, and data are presented as mean ± SD. *p < 0.05; **p < 0.01; ***p < 0.001.

Concomitantly, we performed microarray analysis of RNA expressed in Pogz^+/+^ and Pogz^−/−^ E14.5 FL cells to identify potential target genes and pathways affected by the loss of *Pogz*. We found that 1,062 genes were differentially expressed in Pogz^−/−^ versus Pogz^+/+^ FLs using >1.5-fold change in gene expression as a cutoff (Table S1). Ingenuity Pathway Analysis (IPA) of differentially expressed genes identified the “hematological system and development and function” as a top physiological system affected, and hematological disease” as a top disease and disorder affected (Figure S2F). Differentially expressed genes were linked to erythrocytosis and hereditary persistence of fetal hemoglobin (HPFH) and included *Jak2*, *c-kit*, and *c-myb* (all upregulated) and *Bcl11a* and *Tfrc* (both downregulated) in Pogz^−/−^ FL cells compared to Pogz^+/+^ FL cells (Figure 3A). Taken together, the data from the microarray analysis suggests that loss of *Pogz* may lead to deregulation of erythropoiesis and globin gene expression.

### Loss of *Pogz* Expression Leads to Downregulation of *Bcl11a* and Increased Embryonic β-like Globin Expression

Since *Pogz* is highly expressed in MEPs, and loss of *Pogz* expression affects the expression of genes and pathways required for erythroid development and globin gene expression (Figure 3A), we determined the expression of known transcriptional regulators of erythroid and globin gene expression, including *Klf1*, *Klf2*, *Nfe2*, *Gata1*, *Fog1*, and *Bcl11a*, at E16.5 in Pogz^+/+^ and Pogz^−/−^ FL by real-time qPCR. We found that the expression of *Bcl11a* and *Klf2* was significantly reduced in RNA obtained from E16.5 Pogz^−/−^ FL cells compared to Pogz^+/+^ FL cells, whereas the expression of *Klf1*, *Gata1* and *Fog1* was not significantly different (Figure 3B). We also confirmed a decrease of *Bcl11a* expression at the protein level in E15.5 Pogz^−/−^ FL by western blot analysis (Figure 3C). Since BCL11A is a critical regulator of the switch between fetal and adult globin expression in definitive erythroid cells (Sankaran et al., 2008, 2009, 2010b), we analyzed the expression of the embryonic α- and β-like globins *Hbb*-*y*, *Hbb*-*bh1*, and *Hba*-*x* in E16.5 Pogz^−/−^ FL RNA. We found that the expression of the embryonic globins was significantly upregulated in the Pogz^−/−^ FL compared to Pogz^+/+^ FL (Figure 3D). Thus, expression of *Bcl11a* is decreased and embryonic globin expression is increased in Pogz^−/−^ FL cells, suggesting that Pogz may function to regulate embryonic globin gene expression.

### Persistence of Embryonic Globin Expression Is Intrinsic to *Pogz*^−/−^ Hematopoietic Cells

**Figure 4.**
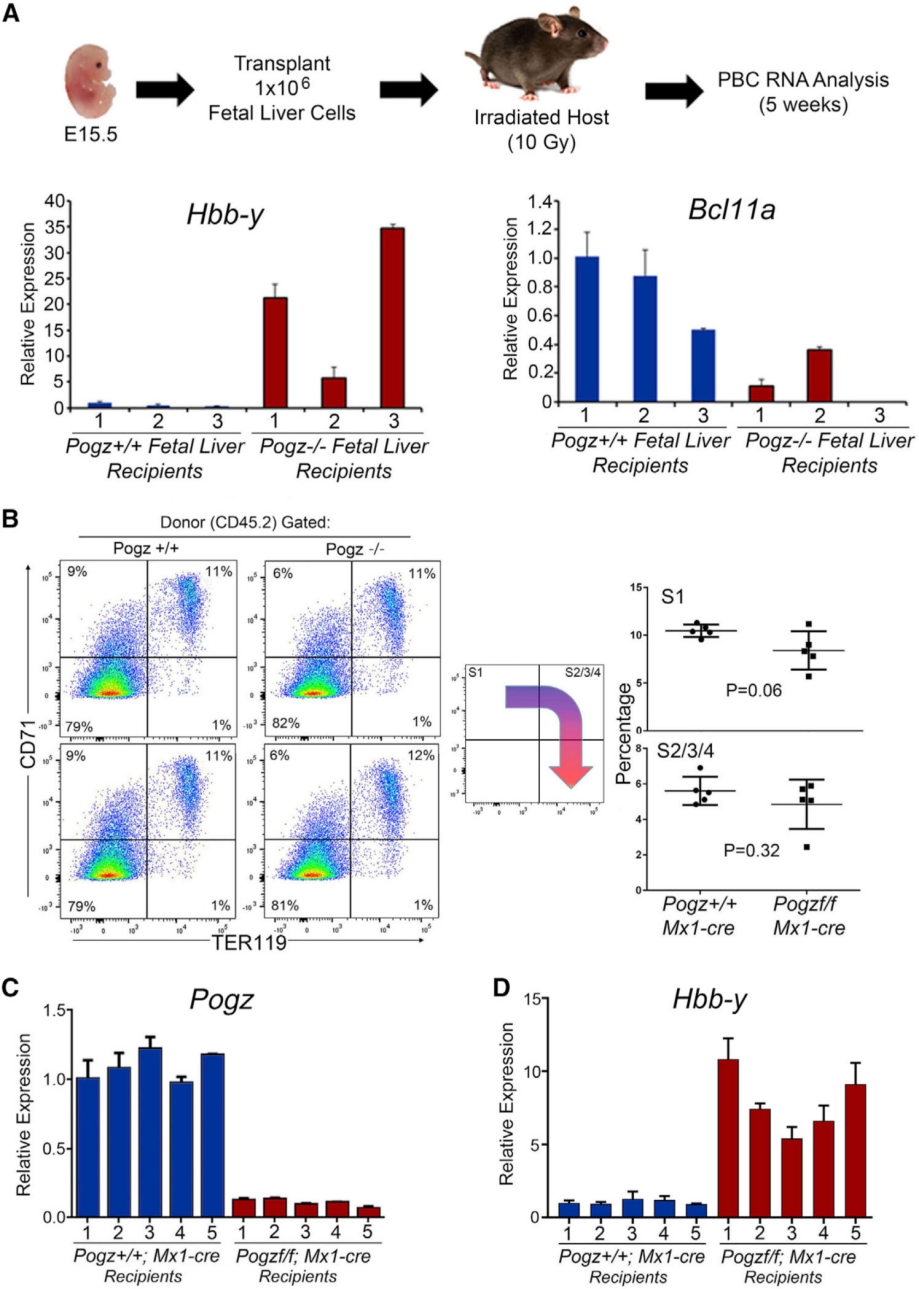
Persistence of Embryonic β-like Globin Expression Is Intrinsic to Pogz^−/−^ Erythroid Cells (A) Summary of FL transplantation experiments. *Hbb*-*y* RNA expression was increased and *Bcl11a* decreased in PBCs obtained from mice 5 weeks after transplantation of Pogz^+/+^ or Pogz^−/−^ FL cells. Gene expression was determined by real-time qPCR and normalized to β-actin expression. Experiments were performed in triplicate, and data are presented as mean ± SD. (B) Representative flow cytometry analysis of CD71 and Ter119 expression in mice transplanted with Pogz^+/+^; Mx1-cre and Pogz^f/f^; Mx1-cre BMCs 12 weeks post-pIpC treatment (n = 5 for each genotype). Gates were set around subsets of differentiating donor (CD45.2^+^ expression) bone marrow (BM) erythroid cells (S0–S5) as previously described (Koulnis et al., 2011). No differences in the percentages of donor-derived erythroid subsets were observed. (C and D) *Pogz* mRNA is not expressed in PBCs from mice transplanted with Pogz^f/f^; Mx1-cre BMCs and treated with pIpC compared to mice transplanted with control Pogz^+/+^; Mx1-cre BMCs (C), while *Hbb*-*y* mRNA expression is significantly increased in PBCs from mice transplanted with Pogz^f/f^; Mx1-cre BMCs (D). Experiments were performed in triplicate. Gene expression was normalized to β-actin expression.

To determine if the increased expression of embryonic globin observed in Pogz^−/−^ FL cells was intrinsic to hematopoietic cells and not an indirect effect due to loss of *Pogz* function in the microenvironment, we transplanted 1 × 10^6^ E15.5 Pogz^+/+^ and Pogz^−/−^ FL cells into lethally irradiated recipient mice (Figure 4A). We found no difference in the number of differentiating erythroid cells at all stages of development (S0–S5) in the bone marrow of mice transplanted with Pogz^−/−^ and Pogz^+/+^ FL cells (Figure S3A). Since CD45 is expressed on more primitive S1, S2, and S3 cells and is decreased during differentiation (S4–S5 cells), we gated on donor-derived CD45.2^+^ cells in mice transplanted with FL cells. Mice transplanted with Pogz^−/−^ FL showed reduced numbers of S1/S2 cells and no difference in the number of S3, S4, or S5 cells compared to mice transplanted with Pogz^−/−^ FL cells, suggesting relatively normal red cell output after transplantation (Figure S3B). We obtained RNA from PBCs 5 weeks after transplantation (short-term reconstitution) and analyzed *Hbb*-*y* and *Bcl11a* expression by real-time qPCR in recipient mice reconstituted with Pogz^+/+^ or Pogz^−/−^ hematopoietic cells. *Hbb*-*y* expression was significantly upregulated in all recipient mice that received Pogz^−/−^ FL cells, while *Hbb*-*y* expression was silenced in recipient mice transplanted with Pogz^+/+^ FL cells (Figure 4A, left panel). The expression of *Bcl11a* was downregulated in mice transplanted with Pogz^−/−^ FL cells in comparison to mice transplanted with Pogz^+/+^ FL cells (Figure 4A, right panel). Furthermore, analysis of *Hbb*-*y* expression 3 months post transplantation (long-term reconstitution) confirmed that *Hbb*-*y* expression remained elevated in mice transplanted with Pogz^−/−^ FL cells, indicating that *Hbb*-*y* expression is not silenced in erythroid cells arising from long-term reconstituting HSPCs (Figure S4A). Collectively, these data suggest that loss of *Pogz* leads to an intrinsic derepression of embryonic β-like globin expression.

While transplantation of Pogz^−/−^ FL cells provides evidence for an intrinsic role of Pogz in regulating embryonic globin gene expression, we sought to confirm this in a model where we could delete *Pogz* in hematopoietic lineage cells in adult mice and limit the potential of non-cell-autonomous effects. First, we bred Pogz conditional mice (Pogz^f/f^) to EpoR-cre transgenic mice (Heinrich et al., 2004) (Pogz^f/f^; EpoR-cre); however, *Pogz* was variably deleted in this model (data not included), which precluded using these mice for further studies. Therefore, we bred Pogz^f/f^ mice to Mx1-cre mice and transplanted BMCs from Pogz^+/+^; Mx1-cre and Pogz^f/f^; Mx1-cre into irradiated recipients to generate chimeric mice (Kühn et al., 1995). Six weeks after bone marrow transplantation (BMT), we treated mice with polyinosinic:polycytidylic acid (pIpC) to delete *Pogz* in hematopoietic cells. Twelve weeks after *Pogz* deletion we analyzed (1) BMCs to confirm that *Pogz* was deleted, (2) PBCs for complete blood cell (CBC) analysis, (3) BMCs for MEP and erythroid development, and (4) PBCs for expression of *Pogz* and *Hbb*-*y* globin. Pogz was efficiently deleted in the chimeric Pogz^f/f^; Mx1-cre transplanted mice (Figure S4B), and results of CBC analysis of mice 12 weeks after deletion of *Pogz* were normal, suggesting that Pogz is not required for normal red cell development in this model (Figure S4C). No differences in donor myeloid and B cell reconstitution were observed in mice transplanted with BMCs that lack *Pogz* (Figure S5A). No difference in the frequency or number of differentiating erythroid cells (S1–S5) was observed in BMCs from Pogz^f/f^; Mx1-cre transplanted mice compared to control transplanted mice (Figure S5B). Further, no significant differences in donor-derived erythroid cell reconstitution were observed in mice transplanted with Pogz^+/+^; Mx1-cre and Pogz^f/f^; Mx1-cre BMC when gated on donor-derived CD45.2^+^ cells, providing additional evidence that loss of *Pogz* does not affect normal adult erythroid cell development (Figure 4B). Since MEPs are restricted progenitors for erythroid cells and platelets and express significant levels of *Pogz*, we examined if loss of *Pogz* affects their development. We found no differences in the number of donor-derived MEPs in BMCs of mice that lack *Pogz* (Figure S5C). In addition, there were no differences in the number of megakaryocyte progenitors (MkPs) and premagakaryocyte/erythroid progenitors in mice transplanted with Pogz^−/−^ and Pogz^+/+^ BMCs (Figure S5D), suggesting that Pogz is not required for the development of erythroid or megakaryocyte progenitors. Finally, we analyzed the expression of *Pogz*, *Hba*-*x*, *Hbb*-*bh1*, and *Hbb*-*y* in PBCs of transplanted mice. Our data confirmed that *Pogz* is not expressed (Figure 4C), while *Hbb*-*y* is expressed in all mice transplanted with Pogz^f/f^; Mx1-cre BMCs (Figure 4D). We also found an upregulation of *Hba*-*x* and *Hbb*-*bh1* expression in some of the animals (Figure S6A–S6D). Collectively, these data demonstrate that Pogz is intrinsically required to repress embryonic β-like globin *Hbb*-*y* in adult red blood cells.

**Figure 5.**
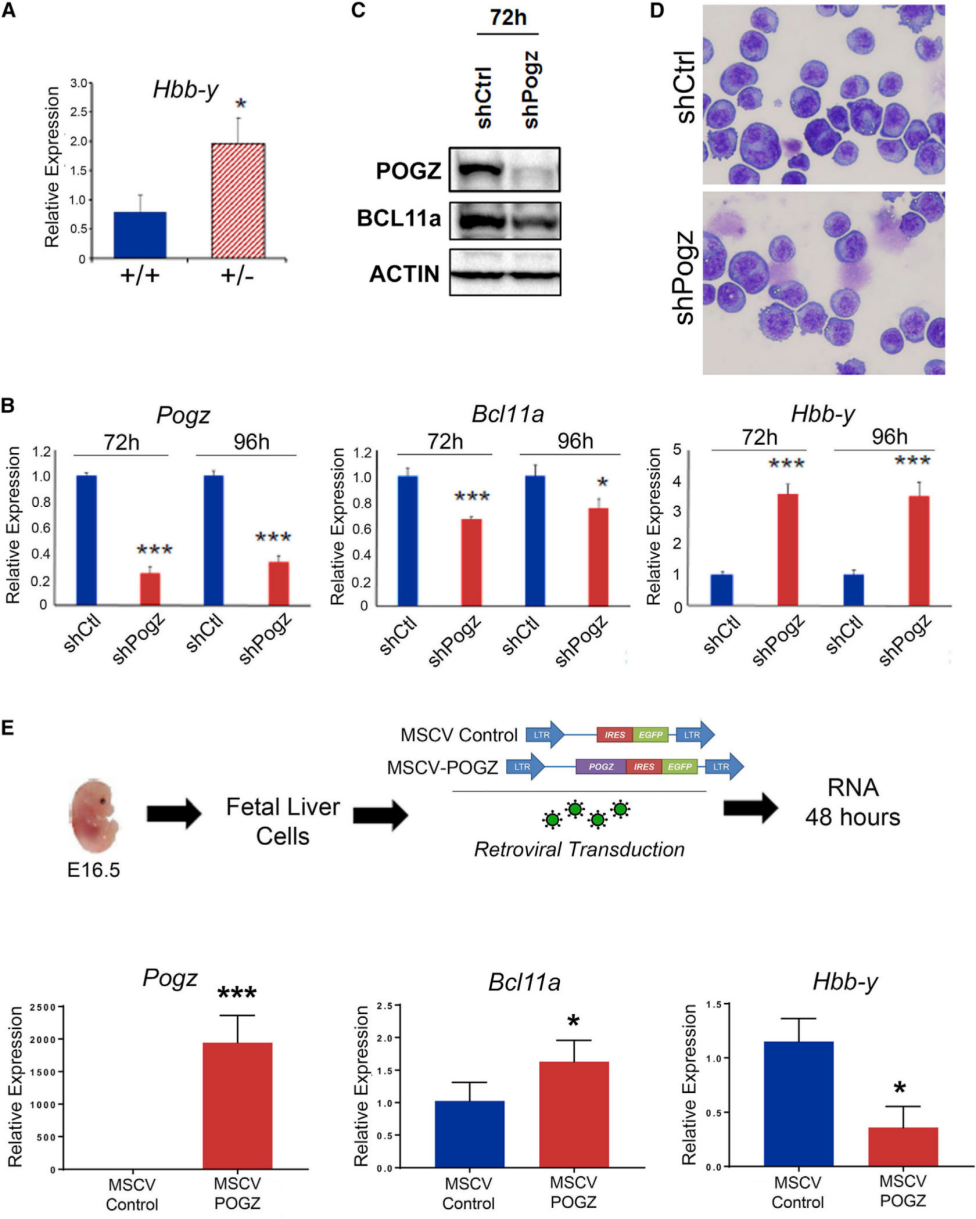
Pogz Regulates the Expression of *Bcl11a* and *Hbb*-*y* in MEL Cells and Fetal Liver Cells (A) *Hbb*-*y* expression is increased in PBC from Pogz^+/−^ compared to Pogz^+/+^ mice. Gene expression was normalized to β-actin expression (n = 5 per group and data are presented as mean ± SD, *p < 0.05). (B) Lentiviral-mediated knockdown of *Pogz* in MEL cells represses *Bcl11a* expression and induces *Hbb*-*y* expression. RNA was harvested from MEL cells 72 and 96 hr after transduction with lentiviral vector expressing *Pogz* shRNA or a control shRNA vector and expression of *Pogz*, *Bcl11a*, and *Hbb*-*y* analyzed by real-time qPCR. Gene expression was normalized to β-actin expression. Experiments were performed in triplicate, and data are presented as mean ± SD. *p < 0.05; ***p < 0.001. (C) Western blot analysis of POGZ, BCL11A, and ACTIN expression following lentiviral-mediated knockdown of *Pogz* in MEL cells. Knockdown was performed with shRNA targeting *Pogz* or a control shRNA vector, and cell lysates were harvested 72 hr post-transduction. (D) Photomicrographs of cytocentrifuge preparations of MEL cells 72 hr after lentiviral-mediated knockdown indicating no effect on cell morphology. (E) Re-expression of *Pogz* in Pogz^−/−^ FL cells induces *Bcl11a* and reduces *Hbb*-*y* RNA expression. Pogz^−/−^ FL cells were harvested at E16.5 and transduced with a control retrovirus or retroviral vector expressing the *Pogz* transgene. RNA was harvested 60 hr post-transduction, and expression of *Hbb*-*y*, *Bcl11a*, and *Pogz* were analyzed by real-time qPCR. Gene expression was normalized to β-actin expression. Experiments were performed in triplicate, and data are presented as mean ± SD. *p < 0.05; ***p < 0.001.

Interestingly, we found that the levels of *Hbb*-*y* expression were significantly increased in PBCs from adult Pogz^+/−^ mice in comparison to Pogz^+/+^ mice, suggesting that Pogz-mediated repression of *Hbb*-*y* expression is dependent on the levels of Pogz expression in adult mice (Figure 5A). Furthermore, since Pogz^+/−^ mice are viable, reproduce, and show no overt phenotype, the data suggest that reducing *Pogz* levels could result in persistence of embryonic globin expression without significantly altering erythroid maturation.

### *Pogz* Negatively Regulates *Hbb*-*y* Expression, in Part through *Bcl11a*

Our data suggest the possibility that POGZ represses *Hbb*-*y* expression, in part by regulating *Bcl11a* expression. To determine if *Pogz* and *Bcl11a* are coexpressed and developmentally regulated in a similar fashion during erythroid differentiation, we analyzed the expression profile of *Pogz* and *Bcl11a* in sorted erythroid cells from the bone marrow of normal C57BL/6J mice (Figure S6E). Our analysis shows that *Pogz* and *Bcl11a* are expressed at similar levels in all erythroid populations (Figures S6F and S6G). As a comparison, *Gata1* and *Klf1* are highly expressed in CD71^HI^Ter119^+^ cells (Figures S6H and S6I). To investigate if Pogz regulates the expression levels of *Bcl11a* and *Hbb*-*y*, we knocked down *Pogz* expression in MEL cells using lentiviral-mediated delivery of *Pogz*-specific small hairpin RNA (shRNA) and analyzed gene expression 72–96 hr post-transduction. The knockdown resulted in significant reduction in *Pogz* mRNA transcripts (Figure 5B) and loss of POGZ protein expression (Figure 5C), which did not affect MEL cell differentiation (Figure 5D) compared to control-treated cells. Loss of *Pogz* expression in MEL cells resulted in decreased *Bcl11a* expression and increased *Hbb*-*y* expression levels (Figures 5B and 5C). In addition, we overexpressed *Pogz* in E16.5 Pogz^−/−^ FL by retroviral transduction and examined *Bclla* and *Hbb*-*y* expression (Figure 5E). Enforced expression of *Pogz* resulted in upregulation of *Bcl11a* expression and repression of *Hbb*-*y* expression. Taken together, these data suggest that Pogz positively regulates *Bcl11a* and represses *Hbb*-*y* expression. Interestingly, overexpression of *Bcl11a* did not reduce *Hbb*-*y* expression in Pogz^−/−^ E16.5 FL cells, which suggests that Pogz may be required for Bcl11a-mediated repression and that there are additional mechanisms by which Pogz represses mouse embryonic β-like globin (Figure S6J).

**Figure 6.**
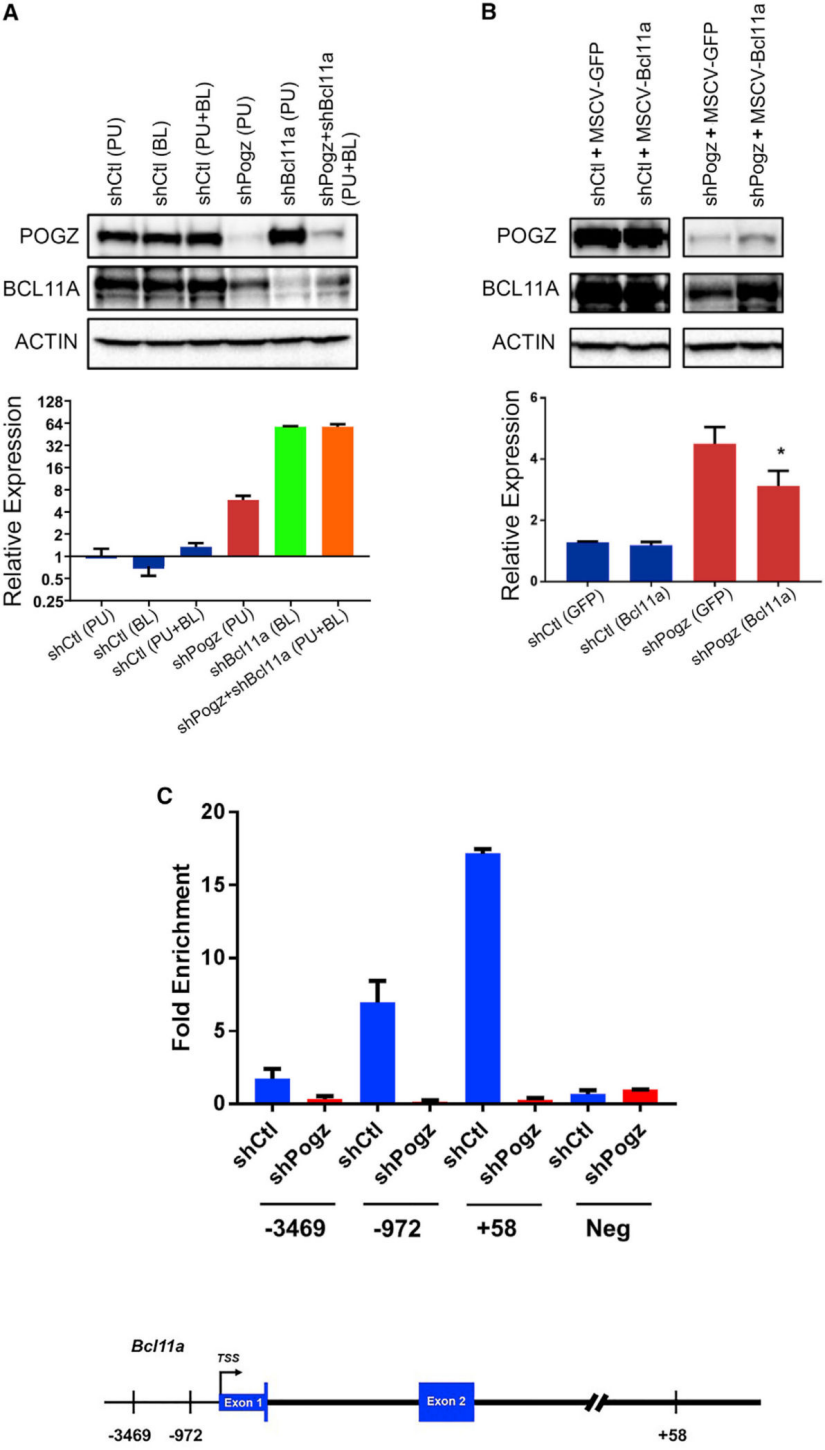
POGZ Regulates *Hbb*-*y* Expression, in Part by Regulating *Bcl11a* Expression (A) Western blot analysis of POGZ, BCL11A, and ACTIN protein levels, and real-time qPCR analysis of *Hbb*-*y* expression following lentiviral-mediated knockdown of *Pogz*, *Bcl11a*, or both *Pogz* and *Bcl11a* in MEL cells. MEL cells were transduced with shRNA targeting *Pogz*, *Bcl11a*, or a control shRNA vector and cell lysates harvested 72 hr post-transduction. Gene expression was normalized to β-actin expression. Experiments were performed in triplicates and data are presented as mean ± SD. PU, puromycin. BL, blasticidin. (B) Western blot analysis of POGZ, BL11A and ACTIN protein levels and real-time qPCR analysis of *Hbb*-*y* expression following lentiviral mediated knockdown of *Pogz* and overexpression of *Bcl11a*. Gene expression was normalized to β-actin expression. Experiments were performed in triplicates and data are presented as mean ± SD. *p < 0.05. (C) ChIP-qPCR analysis demonstrating that POGZ binds to the *Bcl11a* promoter (−972) and enhancer site (+58). POGZ does not bind to a negative control region on chromosome 17. Sheared chromatin was prepared from MEL cells following lentiviral-mediated knockdown of *Pogz*. Knockdown was performed with shRNA targeting *Pogz* or a control shRNA vector, and cell lysates were harvested 72 hr post-transduction. Chromatin was immunoprecipitated with an anti-POGZ antibody and a control antibody. The *Bcl11a* gene indicating sites relative to the transcription start site (TSS) that were examined by ChIP is shown below. Experiments were performed in triplicate, and data are presented as mean ± SD. *p < 0.05; **p < 0.01; ***p < 0.001.

To further examine the requirement for BCL11A in Pogz-mediated regulation of *Hbb*-*y* expression, we performed double knockdown experiments in MEL cells. We found that reducing either *Pogz* or *Bcl11a* expression increases *Hbb*-*y* expression with *Bcl11a* knockdown showing more efficient derepression of *Hbb*-*y* expression (Figure 6A). Knocking down both *Pogz* and *Bcl11a* did not increase *Hbb*-*y* expression above knocking down *Bcl11a* alone (Figure 6A). To examine if *Bcl11a* could rescue the upregulation of *Hbb*-*y* following *Pogz* knockdown, we overexpressed *Bcl11a* in MEL cells transduced with lentiviral vectors that express shPogz. We found that enforced *Bcl11a* expression partially reduces the increase in *Hbb*-*y* expression mediated by Pogz knockdown in MEL cells (Figure 6B), indicating that the *Hbb*-*y* derepression upon loss of *Pogz* is mediated, in part through BCL11A, although additional mechanisms are likely involved in this model.

To test whether POGZ can repress *Hbb*-*y* through direct regulation of *Bcl11a*, we performed chromatin immunoprecipitation (ChIP) assays using MEL cells transduced with control or Pogz shRNA lentiviral vectors. POGZ binds to the *Bcll1a* promoter in MEL cells at −972 and is greatly reduced at −3,469, and POGZ binding to these sites is significantly reduced in cells treated with Pogz shRNA (Figure 6C). POGZ did not show significant binding to a negative control region on mouse chromosome 17. We also examined a recently described enhancer element within intron 2 of the human *BCL11A* gene, which contains three DNase-I-hypersensitive sites at +55, +58, and +62 kb from the transcription start site (Bauer et al., 2013; Canver et al., 2015). The mouse *Bcl11a* gene has orthologous sequences within intron 2 (Bauer et al., 2013). We determined that POGZ binds the orthologous +58 sequences within the enhancer region using ChIP assays (Figure 6C) and that POGZ binding to these sites is reduced when *Pogz* levels are lowered by shRNA. Taken together, our data suggest that POGZ may be a regulator of *Hbb-y* expression by directly or indirectly interacting with the *Bcl11a* promoter and the orthologous *Bcl11a* enhancer elements.

### Reduction of POGZ Expression in Human Proerythroblasts Leads to Increased Expression of Fetal Hemoglobin

**Figure 7.**
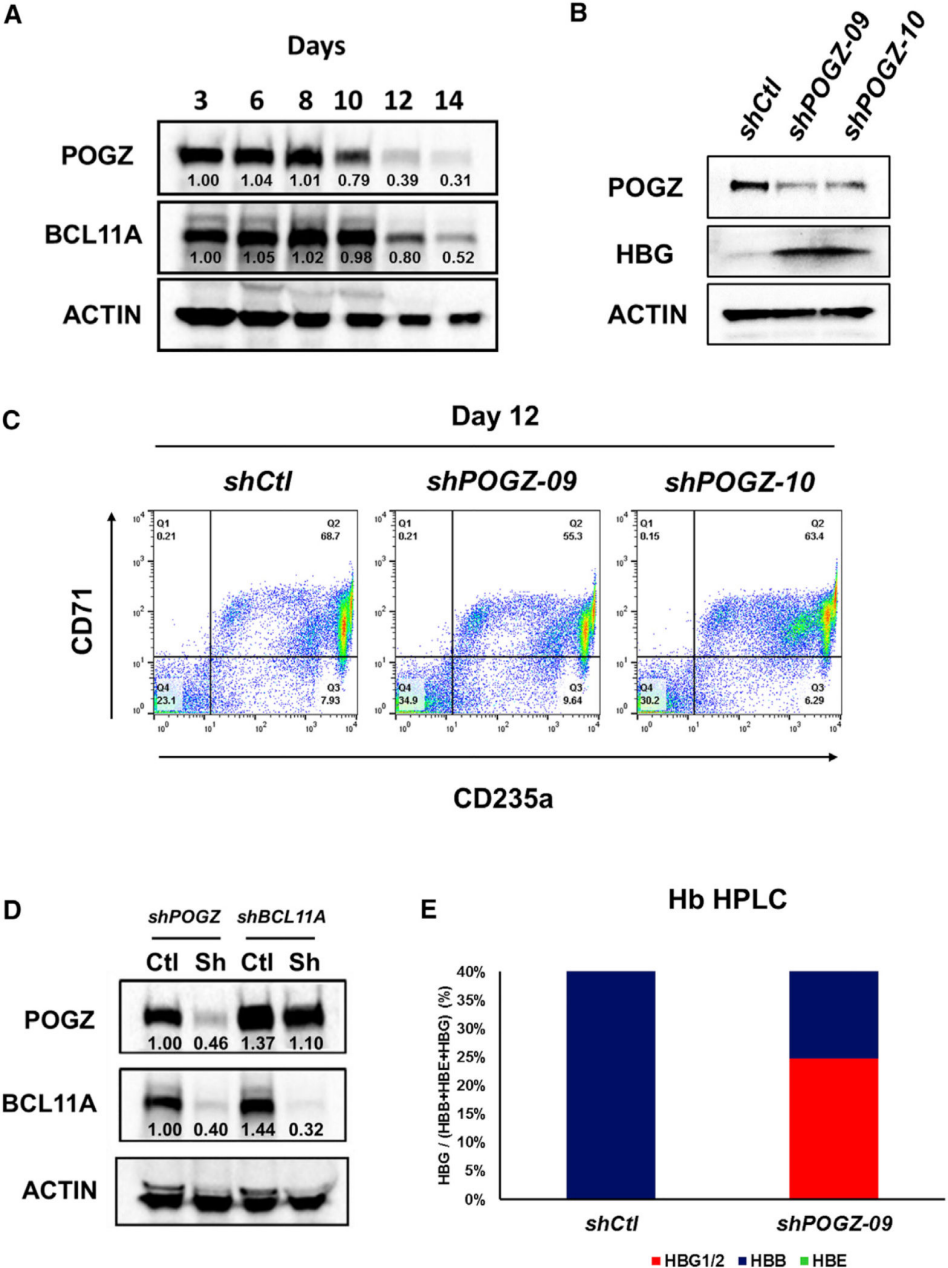
Lentiviral-Mediated Knockdown of *POGZ* Expression in Human Proerythroblasts Decreases *BCL11A* Expression and Increases Fetal Hemoglobin Expression (A) Western blot analysis of POGZ, BCL11A, and ACTIN proteins in cell lysates on days 3–14 of erythroid cultures. Numbers below POGZ and BCL11A bands indicate normalized protein levels in relation to ACTIN. (B) Western blot analysis of POGZ, HBG, and ACTIN protein levels in CD34^+^ cell-derived erythroblasts following lentiviral-mediated knockdown using two separate POGZ shRNA constructs. Knockdown was performed at day 2 of expansion culture with control shRNA or shRNAs targeting *POGZ*, and cell lysates were harvested 10 days post-transduction. (C) Flow cytometry analysis of CD71 and CD235a expression of erythroid cell cultures *in vitro* following lentiviral-mediated knockdown using two separate POGZ shRNA constructs. Knockdown was performed at day 2 of expansion culture with control shRNA or shRNAs targeting *POGZ*, and cells were analyzed 10 days post-transduction. (D) Western blot analysis of POGZ, BCL11A, and ACTIN protein levels in erythroid cell cultures *in vitro* following lentiviral-mediated knockdown of POGZ or BCL11A. Knockdown was performed at day 2 of expansion culture with control shRNA or shRNA targeting *POGZ* or *BCL11A*, and cell lysates were harvested 9 days post-transduction. (E) Representative bar graphs showing fetal hemoglobin (HBG1/2) as a percentage total β-globin (HBB+HBE+HBG1/2) expression following lentiviral-mediated shRNA knockdown of *POGZ*. Erythroblasts were harvested on day 9 post-transduction (day 11 of culture), and the quantities of HBB, HBE, and HBG proteins were determined by HPLC.

To investigate whether POGZ regulates fetal globin expression in human cells, we examined erythroid cells differentiated from adult human CD34^+^ HSPCs in a modified two-phase *in vitro* erythroid culture system as previously described (Migliaccio et al., 2002). We confirmed that the purified CD34^+^ progenitors undergo erythroid differentiation *in vitro* by flow cytometry using CD34, CD45, CD71, and CD235a antibodies (Figure S7A). After 2 days, the cells in these cultures are predominantly primitive CFU-E and proerythroblasts that are CD34^+^CD45^+^CD235a^−^CD71^low^. Most cells undergo further differentiation to more mature CD34^−^CD45^−^CD71^+^CD235a^+^ polychromatic and orthochromatic erythroblasts after 11 days in culture (Figure S7A). In addition, compared to undifferentiated control cells (Figure S7B, left panel), the cell pellets became increasingly red, an indication of increased hemoglobinization and erythroid differentiation (Figure S7B, right panel). We found that POGZ and BCL11A proteins are highly expressed in cells from day 3 cultures (proerythroblasts) and that their expression declines during erythroid differentiation, with little expression after 12–14 days in culture (Figure 7A). To investigate whether POGZ regulates fetal hemoglobin (HBG1/2) expression in human cells, we transduced primary human CD34^+^ cells on day 2 of expansion culture with a control lentiviral shRNA vector or lentiviral shRNA vectors targeting different regions of human *POGZ* coding sequence and measured fetal hemoglobin expression 10 days after transduction by real-time qPCR and western blot. Real-time qPCR analysis showed that both POGZ shRNA constructs significantly reduced *POGZ* transcript levels compared to control shRNA (Figure S7C). *HBG1*/*2* expression was significantly upregulated and *HBB* expression was significantly downregulated, whereas *HBE* and *HBA* expression were marginally affected (Figure S7C). Western blot analysis showed efficient reduction in POGZ protein expression in cultures transduced with POGZ shRNA constructs (Figure 7B), with robust increase in HBG1/2 protein levels, while POGZ or HBG1/2 protein expression was not affected in cultures transduced with control shRNA (Figure 7B). Knockdown of POGZ expression did not affect erythroid differentiation in these cultures, since there was no difference in the extent of erythroid differentiation (CD71^+^CD235a^+^ cells) in cultures treated with control shRNA and POGZ shRNA lentiviral vectors after 12 days (Figure 7C). In agreement with the mouse data above (Figure 5), BCL11A protein levels were significantly reduced upon *POGZ* lentiviral-vector-mediated knockdown, suggesting that POGZ may also regulate *BCL11A* expression in human erythroid cells (Figure 7D). In comparison, POGZ protein levels were minimally affected following lentiviral-vector-mediated *BCL11A* knockdown (Figure 7D). As expected, lentiviral-vector-mediated *BCL11A* knockdown signficantly increased *HBG1*/*2* expression (Figure S7D). HBE expression was also significantly upregulated and HBB and HBA expression significantly downregulated (Figure S7D). Finally, knockdown of *POGZ* results in *HBG1*/*2* protein levels representing roughly 25% of total β-globin, as assessed by high-performance liquid chromatography (HPLC), which is therapeutically relevant (Figure 7E). Taken together, the results suggest that POGZ is a repressor of fetal hemoglobin expression in humans.

## DISCUSSION

In this report, we identified a previously uncharacterized zinc-finger-containing protein, POGZ, which is expressed in mouse and human HSPCs and required to repress embryonic hemoglobin gene expression during normal hematopoietic development. Elevated embryonic globin expression correlated with reduced expression of *Bcl11a*, a known repressor of embryonic β-like globin expression, in Pogz^−/−^ FL cells. We demonstrate, in two different animal models, that red cells develop normally in the absence of *Pogz in vivo* but that the red blood cells (RBCs) show increased embryonic globin expression. Thus, deregulation of embryonic globin expression is intrinsic to Pogz^−/−^ hematopoietic cells, and embryonic globin expression can persist in adult mice after transplantation. Finally, we show that *POGZ* knockdown increases fetal globin expression in primary human erythroblasts, indicating that POGZ also regulates human fetal globin expression, which is the focus of our future studies. These findings are significant since improved therapeutic strategies are needed to treat hereditary globin disorders (Weatherall, 2010). Individuals affected by these diseases have moderate to severe anemia and other serious health issues; however, natural variations that result in HPFH expression are linked to lessening the severity of disease (Sankaran et al., 2010a). Therefore, POGZ may represent a potential therapeutic target to increase fetal globin expression in patients with SCD and β-thalassemia (Bauer et al., 2012).

We found that *Bcl11a* expression is reduced in Pogz^−/−^ FL cells, MEL cells treated with *Pogz* shRNA, and human CD34^+^ progenitors treated with POGZ shRNA, suggesting that Pogz positively regulates *Bcl11a* expression in mouse and human cells. POGZ binding to the *Bcl11a* promoter and a recently identified intron 2 enhancer (Bauer et al., 2013; Canver et al., 2015) suggests that POGZ is directly regulating *Bcl11a* transcription. Future experiments, including ChIP sequencing and electrophoretic mobility shift assays (EMSAs), will determine if this regulation is direct or indirect via interaction with other DNA binding proteins. We also show that BCL11A does not repress embryonic β-like globin *Hbb*-*y* when *Bcl11a* is overexpressed in Pogz^−/−^ FL erythroblasts, suggesting that POGZ may regulate embryonic globin expression by mechanisms other than regulation of *Bcl11a* expression. Since β-actin-Cre-mediated deletion of *Pogz* occurs early in development, the absence of Pogz could affect expression of genes other than *Bcl11a*, which could permanently affect BCL11A’s ability to properly function in these cells. Alternatively, BCL11A may require POGZ expression to repress embryonic globin gene expression. In support of this hypothesis, overexpression of *Bcl11a* in MEL cells transduced with *shPogz* RNA, where *Pogz* expression has been knocked down to 10%–15% of control Pogz expression levels, leads to partial repression of *Hbb*-*y* expression. BCL11A has been the focus of numerous studies to find unique therapeutic targets in SCD and β-thalassemia (Sankaran et al., 2008, 2009, 2010b). It is hypothesized that reduction of BCL11A expression in patients with SCD and β-thalassemia could lead to derepression of fetal hemoglobin, thereby alleviating the symptoms of these disorders (Bauer et al., 2012). The decrease in *Bcl11a* expression and loss of repression of β-like embryonic globin expression upon loss of *Pogz* indicates that POGZ may have the same therapeutic potential.

Mice reconstituted with Pogz^f/f^; Mx-1-cre BMCs survive and show normal development of donor-derived MEP and erythroid lineage cells (S1–S5), as well as lymphoid and myeloid cells, suggesting that inhibiting POGZ function in adults would not have deleterious effects on the host hematopoietic system. However, additional studies are needed to determine whether loss of POGZ function in adults can affect HSC and multipotent progenitor development and function or affect other systems. Interestingly, we found that Pogz^+/−^ mice, which develop normally and show no overt phenotypes, show increased embryonic globin expression levels in PBCs. Importantly, partial reduction of *POGZ* in human erythroblasts also derepressed fetal hemoglobin expression to levels reaching over 25% of total β-like globin. These results suggest that complete ablation of *POGZ in vivo* may not be required to obtain therapeutic benefits. Further *in vitro* and *in vivo* studies are needed to determine if this is feasible.

KLF1 is a master regulator of erythroid development and β-globin expression (Perkins et al., 1995). *Klf1* knockout (KO) mice die in *utero* around E15 due to defects in the differentiation of erythroid cells at the pro-erythroblast stage (Nuez et al., 1995; Perkins et al., 1995; Pilon et al., 2008). KLF1 represses the expression of embryonic globins by upregulating the expression of *Bcl11a* and promotes adult β-globin expression in definitive erythroid cells (Tallack and Perkins, 2013; Zhou et al., 2010). *Klf1* expression was not affected by loss of *Pogz* expression in FL cells. However, global gene expression analysis of Klf1^−/−^ erythroid progenitors demonstrates that *Pogz* is among the significantly downregulated genes (Pilon et al., 2008). Furthermore, analysis of submitted ChIP-sequencing data suggests that KLF1 binds the *POGZ* promoter in human primary erythroid cells, indicating that *POGZ* may be a direct KLF1 target (Su et al., 2013).

Recent evidence suggests that POGZ may have physiological functions in other systems. Specifically, studies analyzing the genetic basis of autism spectrum disorders (ASDs) and intellectual disability have detected inactivating mutations in *POGZ* in some of these patients (Iossifov et al., 2012; Stessman et al., 2016; Tan et al., 2016; White et al., 2016). Our preliminary studies also suggest a function for Pogz in the mammalian neural system, since loss of *Pogz* affects the proliferation of mouse neural progenitor cells in fetal and adult brain (K.O.G., unpublished data). Interestingly, potential disrupting mutations in the *BCL11A* gene have been found in ASDs, and BCL11A has been implicated in neuronal morphogenesis (Iossifov et al., 2012; John et al., 2012). In addition, it was shown in two separate studies that individuals presenting with ASD and developmental delay had common microdeletions of BCL11A rendering them haploinsufficient for the gene. Interestingly, these individuals have elevated expression of fetal hemoglobin (Basak et al., 2015; Funnell et al., 2015). These data suggest that BCL11A and POGZ could function within the same regulatory networks in the neural system.

In summary, our data show that POGZ is essential for normal embryonic development and that loss of the gene leads to deregulation of embryonic globin expression, in part through *Bcl11a*. Reduction of *POGZ* expression in erythroid cells could have therapeutic implications in SCD and β-thalassemia.

## EXPERIMENTAL PROCEDURES

### Mice

Conventional Pogz^−/−^ mice and conditional Pogz^f/f^, and Pogz^f/f^; Mx1-cre mice were generated as described in Supplemental Experimental Procedures. Female mice aged 8–12 weeks were used as recipients for all transplantation experiments. Mice were housed, fed, and handled in accordance with the National Institutes of Health guidelines for animal care and use and the *Guide for the Care and Use of Laboratory Animals, 8th Edition*. All mouse experiments were reviewed and approved by the Institutional Animal Care and Use Committee of the National Cancer Institute at Frederick, which is accredited by Association for Assessment and Accreditation of Laboratory Animal Care (AAALAC) International.

### Real-Time qPCR

Analysis of *POGZ* expression in KG1 cells by real-time qPCR was performed as described previously (Gudmundsson et al., 2007). Globin expression was analyzed in CD34^+^ HSPC-derived human erythroblasts using Taqman assays. For mouse FL cells, PBC and BMC RNA was isolated and real-time qPCR analysis performed in triplicate using Power SYBR Green PCR Master Mix (Life Technologies) and a 7500 Real-Time PCR System (Life Technologies) as previously described (Oakley et al., 2012). The ΔCt method was used to calculate relative changes in gene expression. Primer sequences are presented in Supplemental Experimental Procedures.

### Flow Cytometry

Single-cell suspensions were prepared from Pogz^+/+^ or Pogz^−/−^ FLs or from BMCs and PBCs from animals transplanted with Pogz^+/+^ or Pogz^−/−^ FL cells or Pogz^+/+^; Mx1-cre or Pogz^f/f^; Mx1-cre BMCs. Cells were incubated with the antibodies described in Supplemental Experimental Procedures and then analyzed by FACS-CantoII (BD Biosciences), and data were analyzed using FlowJo software (Tree Star).

### FL and BMC Transplantations

FL cells were harvested from E14.5–E16.5 Pogz^+/+^ and Pogz^−/−^ embryos, and BMCs were isolated from adult Pogz^+/+^; Mx1-cre and Pogz^f/f^; Mx1-cre mice and then transplanted as described in Supplemental Experimental Procedures using standard methodologies (Gudmundsson et al., 2012, 2014).

### Statistical Analysis

Statistical analysis was performed using GraphPad Prism (GraphPad Software). An unpaired Student’s t test was used to calculate statistical significance. Results were considered significant if p < 0.05. Results are presented as the mean ± SD.

## DATA AND SOFTWARE AVAILABILITY

The accession number for the microarray data reported in this paper is GEO: GSE113503.

## Supplementary Material

Supplemental

Table S1

Table S2

## Highlights

- *Pogz* is highly expressed in mouse megakaryocyte erythroid progenitors
- POGZ is required to repress murine embryonic β-like globin during erythropoiesis
- Pogz^+/−^ mice develop normally and show elevated embryonic β-like globin expression
- *POGZ* knockdown decreases *BCL11A* and increases fetal globin expression in human cells
