# Supplementary material for: POGZ Is Required for Silencing Mouse Embryonic β-like Hemoglobin and Human Fetal Hemoglobin Expression
Source: Cell Rep. Author manuscript; Available in PMC 2020 Jun 18. (PMC7301966; doi:10.1016/j.celrep.2018.05.043)
Supplement: Supplemental [file NIHMS1590638-supplement-Supplemental.pdf]

Cell Reports, Volume *23*

Supplemental Information

POGZ Is Required for Silencing

Mouse Embryonic β-like Hemoglobin

and Human Fetal Hemoglobin Expression

Bjorg Gudmundsdottir, Kristbjorn O. Gudmundsson, Kimberly D. Klarmann, Satyendra K. Singh, Lei Sun, Shweta Singh, Yang Du, Vincenzo Coppola, Luke Stockwin, Nhu Nguyen, Lino Tessarollo, Leifur Thorsteinsson, Olafur E. Sigurjonsson, Sveinn Gudmundsson, Thorunn Rafnar, John F. Tisdale, and Jonathan R. Keller

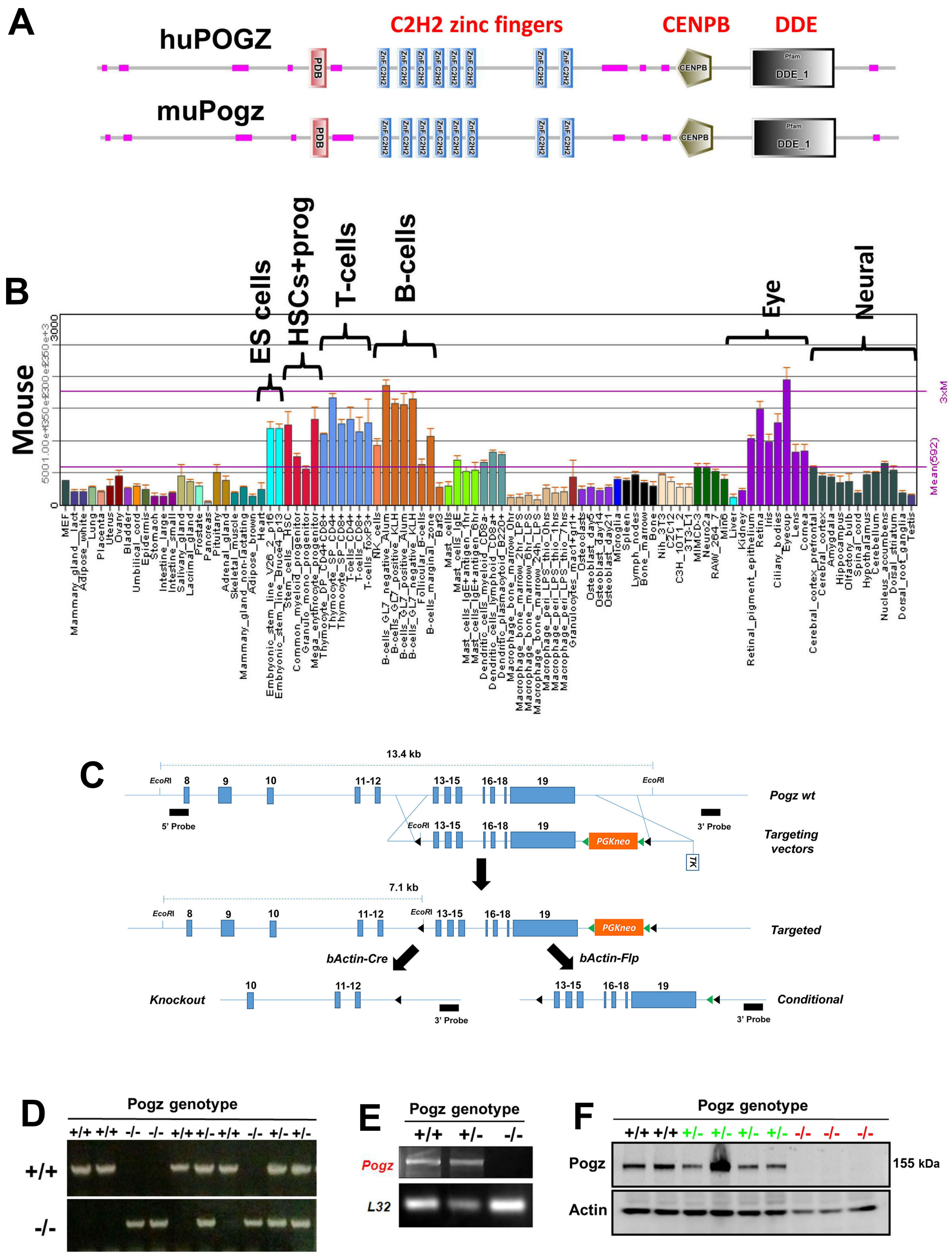
A
huPOGZ
muPogz
C2H2 zinc fingers
CENPB
DDE
PDB
ZnF C2H2
Pfam
DDE_1
B
Mouse
ES cells
HSCs+prog
T-cells
B-cells
Eye
Neural
3xM
Mean(592)
C
13.4 kb
EcoRI
5' Probe
3' Probe
Pogz wt
Targeting vectors
PGKneo
TK
7.1 kb
Targeted
bActin-Cre
bActin-Flp
Knockout
Conditional
D
Pogz genotype
+/+
-/-
E
Pogz genotype
Pogz
L32
F
Pogz genotype
Pogz
Actin
155 kDa

Figure S1

Figure S1. Pogz domain structure, expression profile, and generation of mice with targeted *Pogz* gene deletion, Related to Figure 2. (**A**) Summary of human and mouse POGZ proteins using SMART (Simple Modular Architecture Research Tool) analysis (http://smart.embl-heidelberg.de/) demonstrating identical domain structure with the presence of multiple C2H2 zinc fingers, a CENPB-type domain and a DDE domain. Regions of low complexity are represented in magenta. (**B**) *Pogz* expression profile in mouse primary cells, tissues, and cell lines. Probeset ID mouse: 1455046_a_at. Data were obtained from the BioGPS gene expression database (**http://biogps.org**). (**C**) Schematic overview of the *Pogz* targeting strategy. The exons are depicted as blue boxes, loxp sites as black arrowheads, and Frt sites as green arrowheads. The *Pogz* gene spans approximately 26.4 kb and contains 19 exons. To delete as many functional protein domains as possible, we flanked a 4.13 kb region on the 3´end of the *Pogz* gene with loxp sites. This region contains exons 13-19 encoding three zinc fingers, the CENPB domain, the DDE domain and a part of the 3' untranslated region. The conditional knockout vector for *Pogz* was constructed using the recombineering method and is described in the supplemental methods (Liu et al., 2003). For generating homozygous Pogz−⁄− embryos, heterozygous Pogz+/- mice were intercrossed and pregnant mice harvested at different time points. (**D**) Genotyping of E15.5 Pogz embryos using primer pairs specifically designed to generate wild type or knockout PCR products. (**E**) RT-PCR analysis of the full length Pogz coding sequence using RNA isolated from the brains of E14.5 Pogz+/+, Pogz+/- and Pogz-/- embryos. (**F**) Western blot analysis of whole cell lysates generated from E15.5 Pogz+/+, Pogz+/- and Pogz-/- fetal brains.

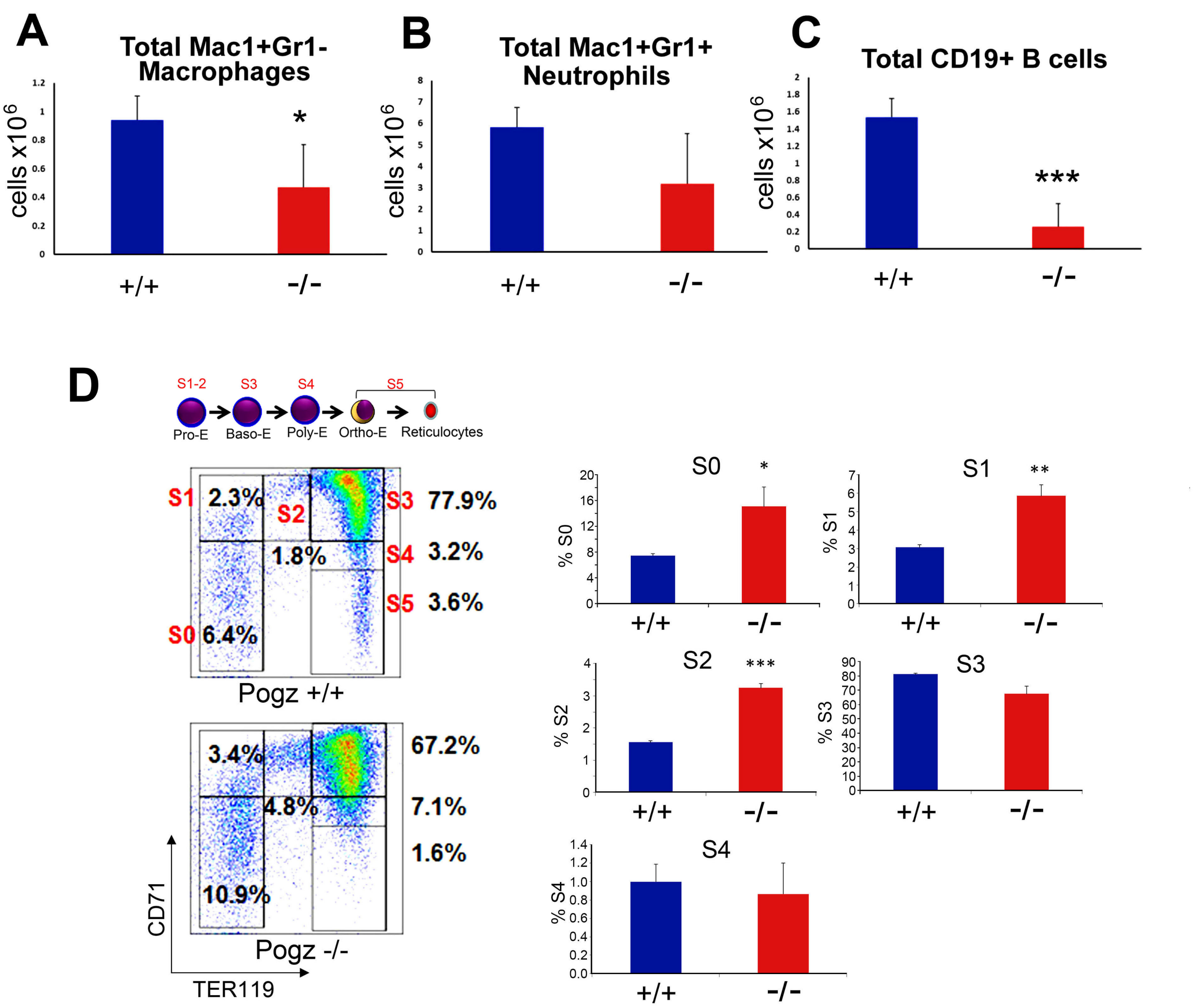

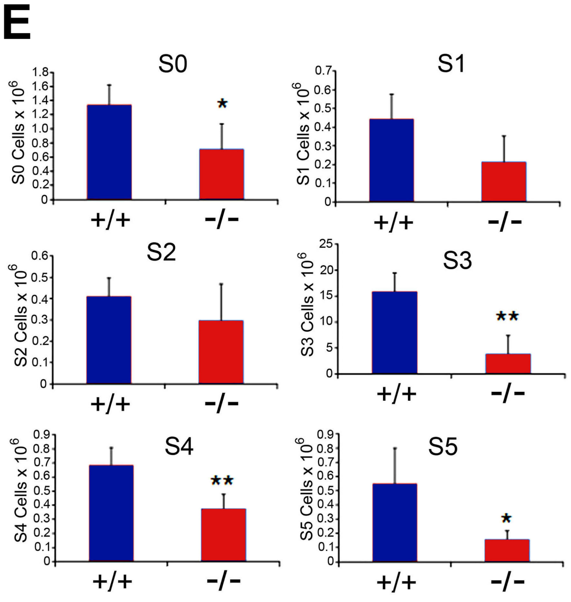

F

Functions	p-Value	Molecules
Treatment-related acute myeloid leukemia	1,84E-03	Idh2, Jak2, Kit, Npm1
Erythrocytosis	5,62E-03	Add2, ApoE, Arid4a, Egln1, Idh2, Il1rl1, Jak2, Socs3
Diffuse lymphoma	5,62E-03	B2m, Cflar, Crebbp, Ctnnb1, Kit, Mll2, Myc, PolB, Tcf3, Ywhaz
Diffuse large B-cell lymphoma	6,05E-03	B2m, Crebbp, Ctnnb1, Kit, Mll2, Myc, Ywhaz
Chronic myelomonocytic leukemia	1,05E-02	Cbl, Idh2, Jak2, Npm1, Srsf2
Megakaryocytosis of bone marrow	1,26E-02	Crebbp, Myb
Diffuse B-cell lymphoma	1,80E-02	B2m, Cflar, Crebbp, Ctnnb1, Kit, Mll2, Myc, Ywhaz
Acute lymphocytic leukemia	1,82E-02	Bax, Crebbp, Jak1, Jak2, Kit, Myc, PolB, Ppat, Rrm1, Runx1t1, Slc19a1, Slc6a3, Tax1bp1
Precursor B-cell acute lymphoblastic leukemia	1,86E-02	Crebbp, Jak1, Jak2, Kit, Runx1t1
Hereditary persistence of fetal hemoglobin	2,40E-02	Bcl11a, Hbb-bh1, Myb, Myc
Myelodysplastic syndrome	2,43E-02	Idh2, Jak2, Kit, Myc, Npm1, Pdia3, Rpl13, Rpl13a, Srsf2
Natural killer cell lymphoma	2,44E-02	Ctnnb1, Kit, Myc
Leukemogenesis	3,33E-02	Jak2, Myb, Myc, Runx1t1

Figure S2

Figure S2. Reduced output of hematopoietic cells in Pogz-/- fetal livers, Related to Figure 2 and Figure 3. Total number of (A) macrophages (Mac1+Gr1-), (B) neutrophils (Mac1+Gr1+) and (C) B cells (CD19+) in E15.5 Pogz+/+ and Pogz-/- fetal livers. N=3 for each genotype. Data are presented as mean ± SD. * $P < 0.05$. *** $P < 0.001$ and are representative of three separate experiments. (D) Schematic overview of differentiating red blood cells and corresponding flow cytometry gates for FL cells. Representative flow cytometry analysis of CD71 and Ter119 expression in E15.5 Pogz+/+ and Pogz-/- fetal liver (FL) cells (N=4 for each genotype). Gates were set around subsets of differentiating FL erythroid cells (S0-S5) as previously described (Koulnis et al., 2011). The frequencies for all cell populations are shown (N= 4 mice/group), and are representative of three separate experiments. Pogz-/- FLs show increased percentages of S0, S1 and S2 cells. (E) Pogz-/- FLs show a reduction in the total number of more differentiated erythroid cells (S3-S5). Data are presented as mean ± SD. * $P < 0.05$; ** $P < 0.01$, and are representative of three separate experiments. (F) Loss of *Pogz* affects expression of genes linked to hematological disease. Genes denoted with green color are downregulated in Pogz-/- fetal livers. Genes denoted with red color are upregulated in Pogz-/- fetal livers.

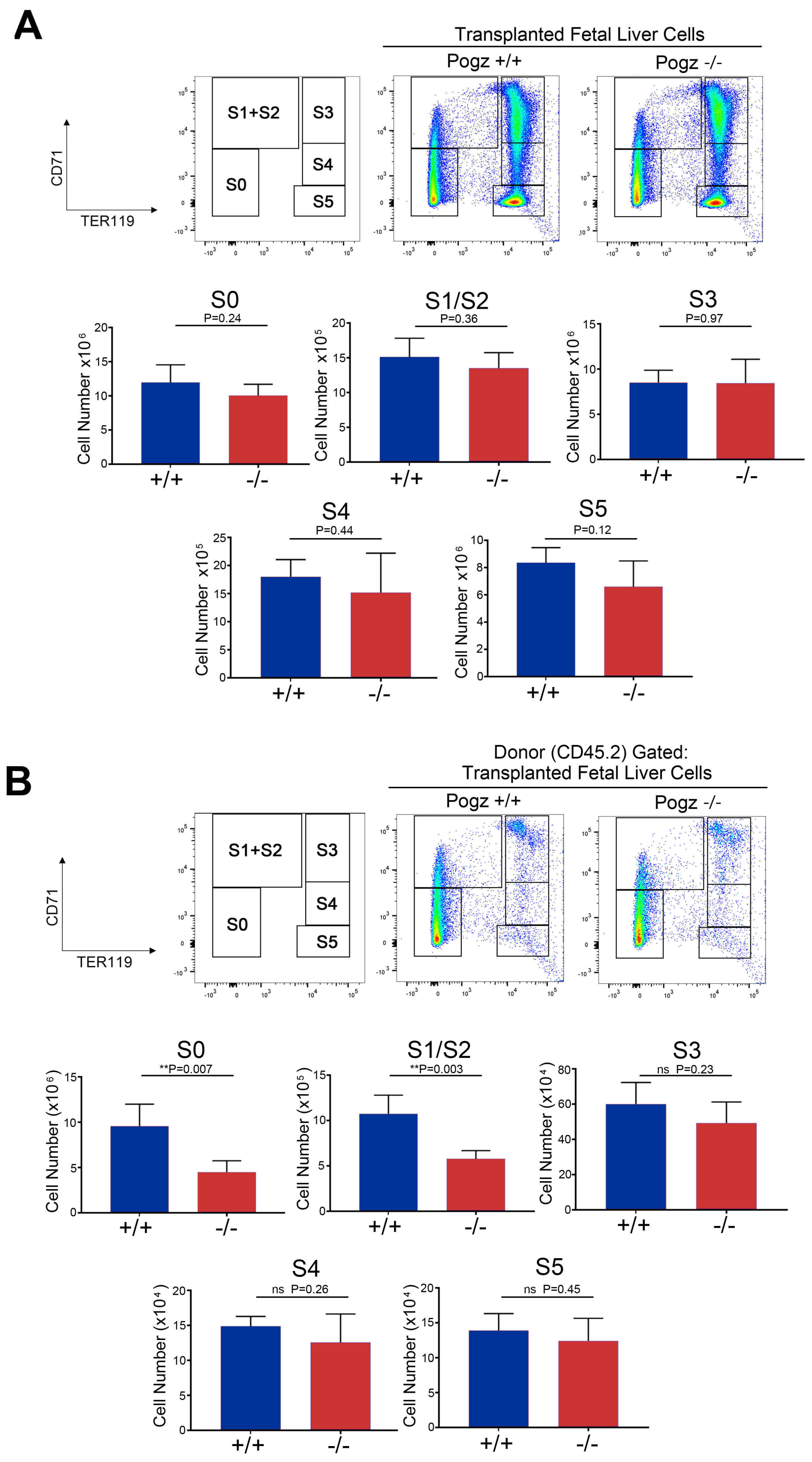
A
Transplanted Fetal Liver Cells
Pogz +/+
Pogz -/-
CD71
TER119
S1+S2
S3
S4
S0
S5
S0
P=0.24
S1/S2
P=0.36
S3
P=0.97
S4
P=0.44
S5
P=0.12
Cell Number x10⁶
Cell Number x10⁵
+/+
-/-
B
Donor (CD45.2) Gated:
Transplanted Fetal Liver Cells
Pogz +/+
Pogz -/-
S0
**P=0.007
S1/S2
**P=0.003
S3
ns P=0.23
S4
ns P=0.26
S5
ns P=0.45
Cell Number (x10⁶)
Cell Number (x10⁵)
Cell Number (x10⁴)

Figure S3

Figure S3. Erythroid development in mice transplanted with Pogz-/- fetal liver cells, Related to Figure 4. Representative flow cytometry analysis of CD71 and Ter119 expression in recipients of E15.5 Pogz+/+ (N=5) and Pogz-/- FL cells (N=4) 4 months after transplantation. Gates were set around subsets of differentiating BM erythroid cells (S0-S5) as previously described (Koulnis et al., 2011). (**A**) Flow cytometry analysis of total BMCs from recipient mice and (**B**) flow cytometry analysis of CD45.2+ donor-derived BMCs. Data are presented as mean ± SD. ** $P < 0.01$, and are representative of two separate experiments.

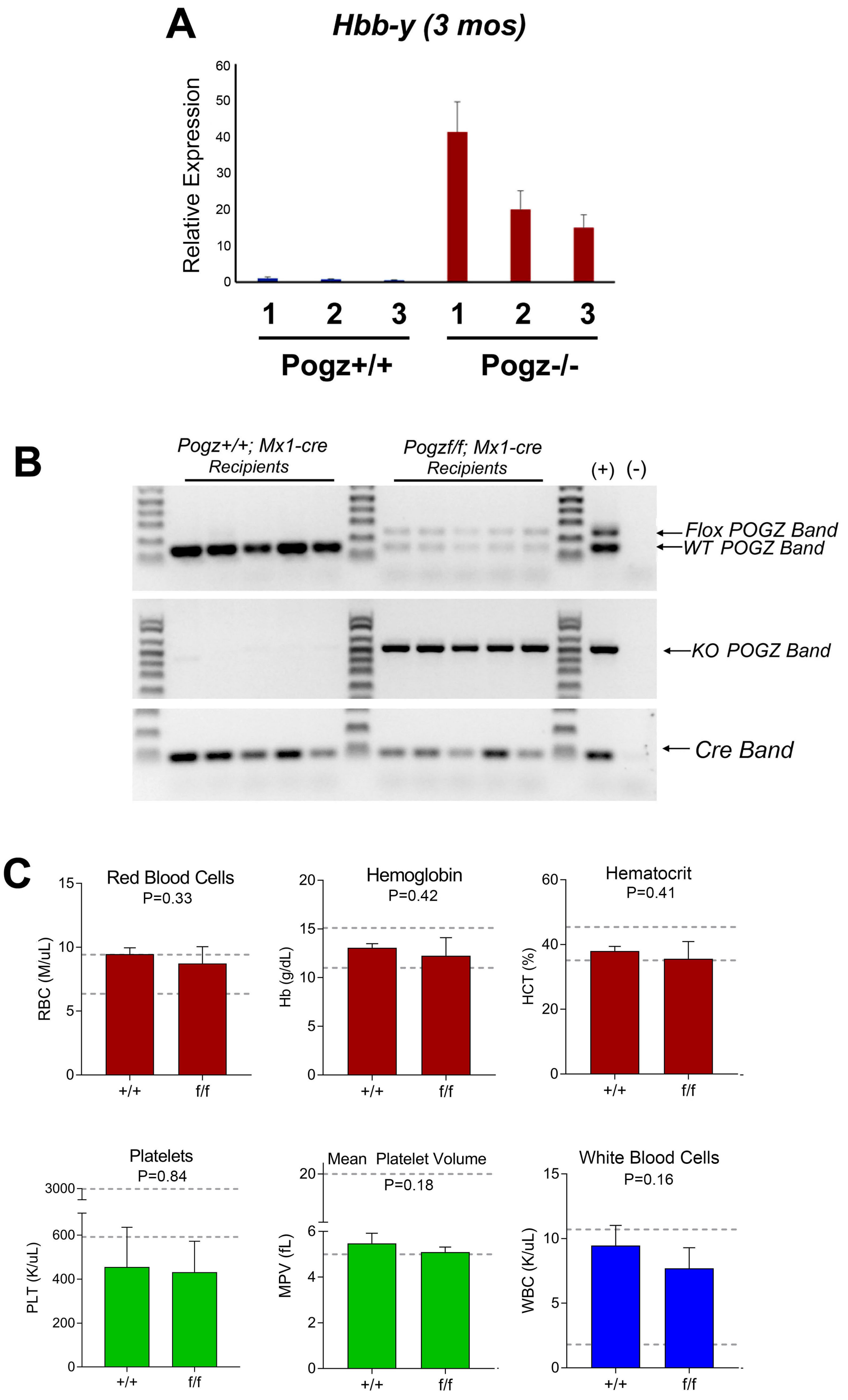

A
Hbb-y (3 mos)
Relative Expression
60
50
40
30
20
10
0
1
2
3
1
2
3
Pogz+/+
Pogz-/-
B
Pogzf/f; Mx1-cre Recipients
Pogz+/+; Mx1-cre Recipients
(+)
(-)
Flox POGZ Band
WT POGZ Band
KO POGZ Band
Cre Band
C
Red Blood Cells
P=0.33
RBC (M/uL)
+/+
f/f
Hemoglobin
P=0.42
Hb (g/dL)
Hematocrit
P=0.41
HCT (%)
Platelets
P=0.84
PLT (K/uL)
Mean Platelet Volume
P=0.18
MPV (fL)
White Blood Cells
P=0.16
WBC (K/uL)

Figure S4

Figure S4. Persistence of fetal globin expression and analysis of PBCs in transplant recipient mice, Related to Figure 4. (A) Persistence of embryonic *Hbb-y* globin expression in mice transplanted with Pogz-/- FL cells. RNA was purified from PBCs isolated from mice 3 months after transplantation with Pogz+/+ and Pogz-/- FL cells, and *Hbb-y* expression analyzed by real-time RT-qPCR. Gene expression was normalized to β-actin expression. Experiments were performed in triplicate and data are presented as mean ± SD, and representative of two separate experiments. (B) PBCs were isolated from mice transplanted with Pogz+/+; Mx1-cre and Pogzf/f; Mx1-cre 12 weeks after treatment with pIpC. DNA was extracted from PBCs and individual recipient mice were genotyped using primers that specifically amplify Pogz+/+ and Pogz-/- DNA by PCR followed by gel electrophoresis. (C) CBC analysis of PBCs obtained from mice transplanted with Pogz+/+; Mx1-cre or Pogzf/f; Mx1-cre or BMC 12 weeks post pIpC injections (N=5 for each genotype). No significant differences in red blood cell counts, hemoglobin concentration, hematocrit (%), platelets, mean platelet volume, and white blood cell counts were observed in mice transplanted with Pogz+/+; Mx1-cre BMC or Pogzf/f; Mx1-cre BMCs. Data are presented as mean ± SD. * P = 0.01, and are representative of three separate experiments.

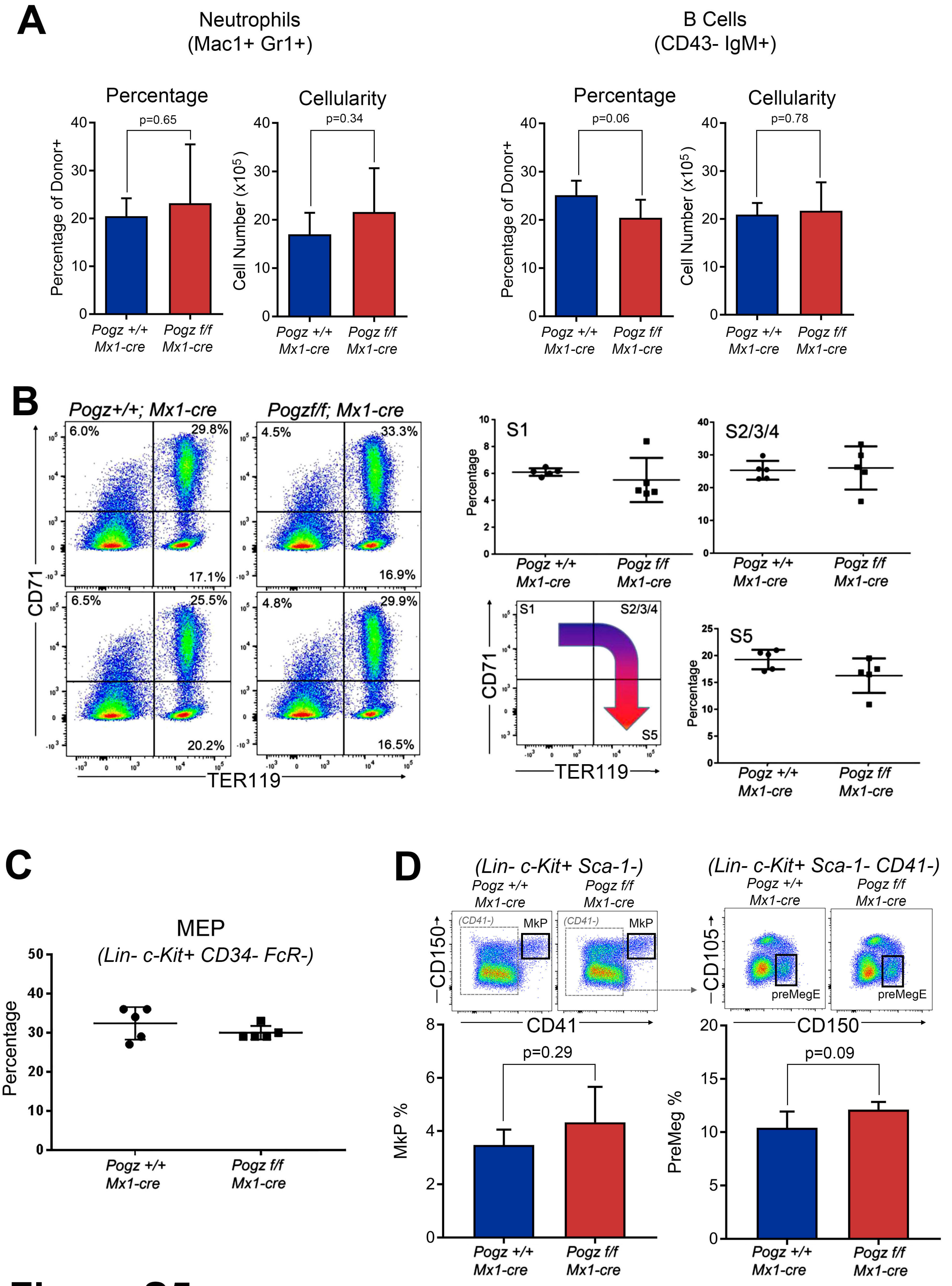
A
Neutrophils
(Mac1+ Gr1+)
B Cells
(CD43- IgM+)
Percentage
Cellularity
p=0.65
p=0.34
p=0.06
p=0.78
Percentage of Donor+
Cell Number (x10^5)
Pogz +/+ Mx1-cre
Pogz f/f Mx1-cre
B
Pogz+/+; Mx1-cre
Pogzf/f; Mx1-cre
6.0%
29.8%
17.1%
4.5%
33.3%
16.9%
6.5%
25.5%
20.2%
4.8%
29.9%
16.5%
CD71
TER119
S1
S2/3/4
S5
Percentage
C
MEP
(Lin- c-Kit+ CD34- FcR-)
Percentage
D
(Lin- c-Kit+ Sca-1-)
(Lin- c-Kit+ Sca-1- CD41-)
(CD41-)
MkP
CD150
CD41
CD105
preMegE
p=0.29
p=0.09
MkP %
PreMeg %

Figure S5

Figure S5. Analysis of hematopoietic development in mice transplanted with Pogz+/+; Mx1-cre or Pogzf/f; Mx1-cre BMC, Related to Figure 4. (**A**) No difference in neutrophil or B cell development was observed in mice transplanted with Pogz+/+; Mx1-cre or Pogzf/f; Mx1-cre BMCs 12 weeks after pIpC treatment. The frequency and total number of donor neutrophils and mature B cells for individual mice is presented. (**B**) Representative flow cytometry analysis of CD71 and Ter119 expression in differentiating erythroid cells (S0-S5) in mice transplanted with Pogz+/+; Mx1-cre or Pogzf/f; Mx1-cre BMCs 12 weeks after pIpC treatment. No difference in the frequency of differentiating erythroid cells in S1, combined S2/3/4 or S5 gates was observed. (**C**) BMCs were stained with lineage markers, c-Kit, Sca1, CD34 and Fcrγ to analyze the frequency of MEPs by flow cytometry. No difference in the frequency of donor MEPs was observed in mice transplanted with Pogz+/+; Mx1-cre or Pogzf/f; Mx1-cre BMCs 12 weeks after pIpC treatment. **(D)** Frequency of donor Mk and preMegE progenitors in mice transplanted with Pogz+/+; Mx1-cre or Pogzf/f; Mx1-cre BMC 12 weeks after pIpC injections. BMCs were stained with lineage markers, c-Kit, Sca1, CD41, CD150, and CD105 and analyzed by flow cytometry. The data presented in **A-D** are representative of at least two separate experiments with five mice analyzed per group.

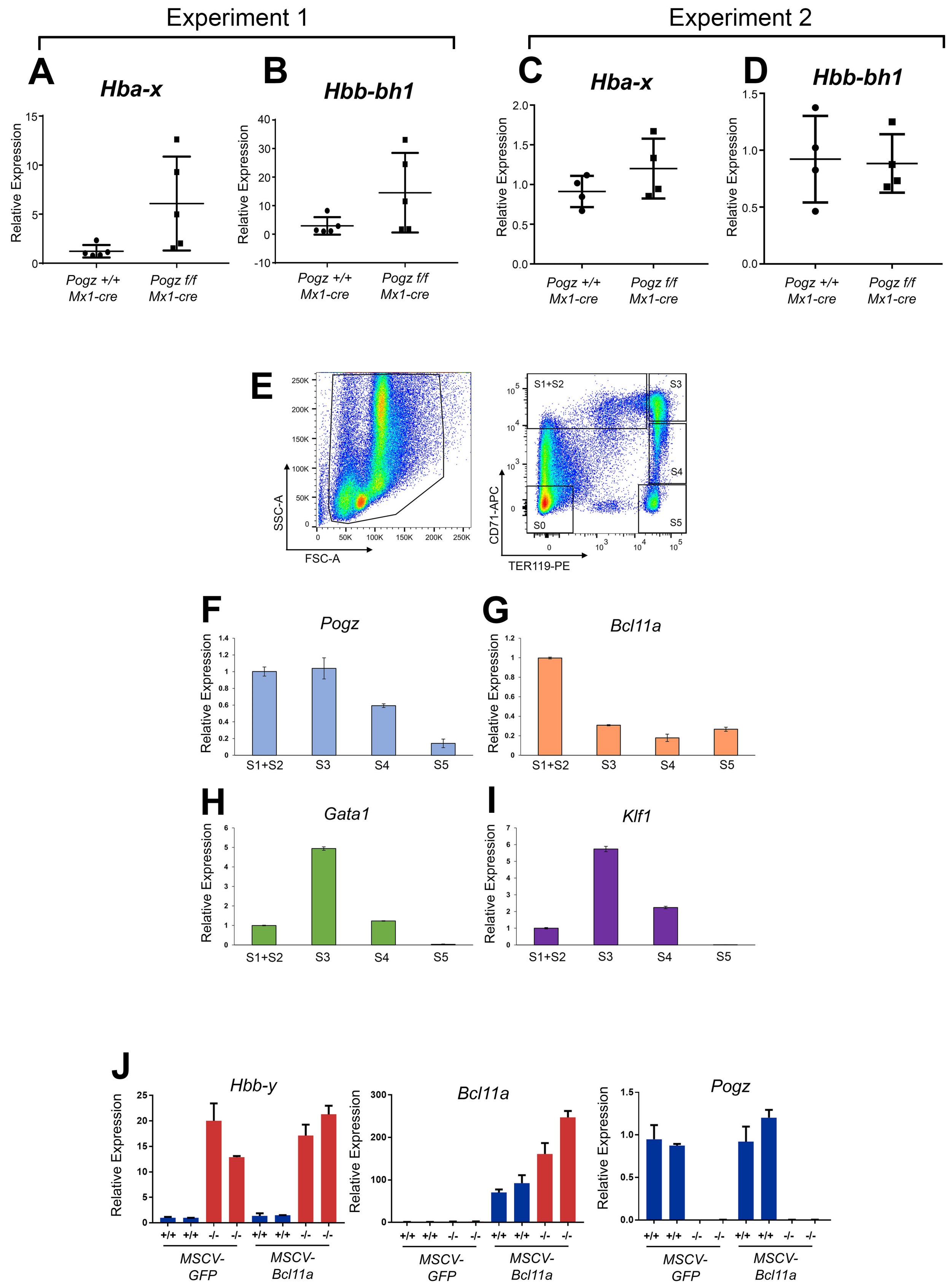
Experiment 1
Experiment 2
A
Hba-x
B
Hbb-bh1
C
Hba-x
D
Hbb-bh1
Relative Expression
Pogz +/+ Mx1-cre
Pogz f/f Mx1-cre
E
SSC-A
FSC-A
CD71-APC
TER119-PE
S1+S2
S3
S4
S5
S0
F
Pogz
G
Bcl11a
H
Gata1
I
Klf1
J
Hbb-y
Bcl11a
Pogz
+/+
-/-
MSCV-GFP
MSCV-Bcl11a

Figure S6

Figure S6. Expression and regulation of hemoglobin and transcription factor expression in mice that lack *Pogz* expression, Related to Figure 4 and Figure 5. (A-D) *Hba-x* and *Hbb-bh1* expression was elevated is some mice transplanted with Pogzf/f; Mx1-cre BMCs. BMCs were harvested from recipient mice transplanted with Pogz+/+; Mx1-cre or Pogzf/f; Mx1-cre BMC 12 weeks post pIpC treatment, and expression of *Hba-x and Hbb-bh1* was analyzed by real-time qRT-PCR (N=5 per genotype) in two separate experiments. Gene expression was normalized to *β-actin* expression. **(E-I)** *Pogz, Bcl11a, Gata1* and *Klf1* RNA expression was determined in FACS sorted erythroid cells from the bone marrow of normal mice by qRT-PCR. Bone marrow erythroid cells from C57BL/6J mice were stained with Ter119 and CD71 antibodies and sorted into 4 populations (Ter119-CD71+ {S1-2}, Ter119+CD71^{HI} {S3}, Ter119+CD71^{Med} {S4}, and Ter119+CD71- {S5}) using the gates defined. **(F)** *Pogz* and **(G)** *Bcl11a* show similar RNA expression profiles throughout erythroid differentiation, whereas **(H)** *Gata1* and **(I)** *Klf1* RNA expression is predominantly in Ter119+CD71^{HI} (S3) cells. **(J)** Overexpression of *Bcl11a* in E16.5 Pogz-/- FL cells does not repress *Hbb-y* expression. E16.5 Pogz+/+ and Pogz-/- FL erythroid progenitors (N=2 each genotype) were transduced with a control retroviral vector or a *Bcl11a* retroviral vector, and harvested 60 hrs post transduction for qRT-PCR analysis of *Hbb-y*, *Pogz* and *Bcl11a* expression. Gene expression was normalized to *β-actin* expression.

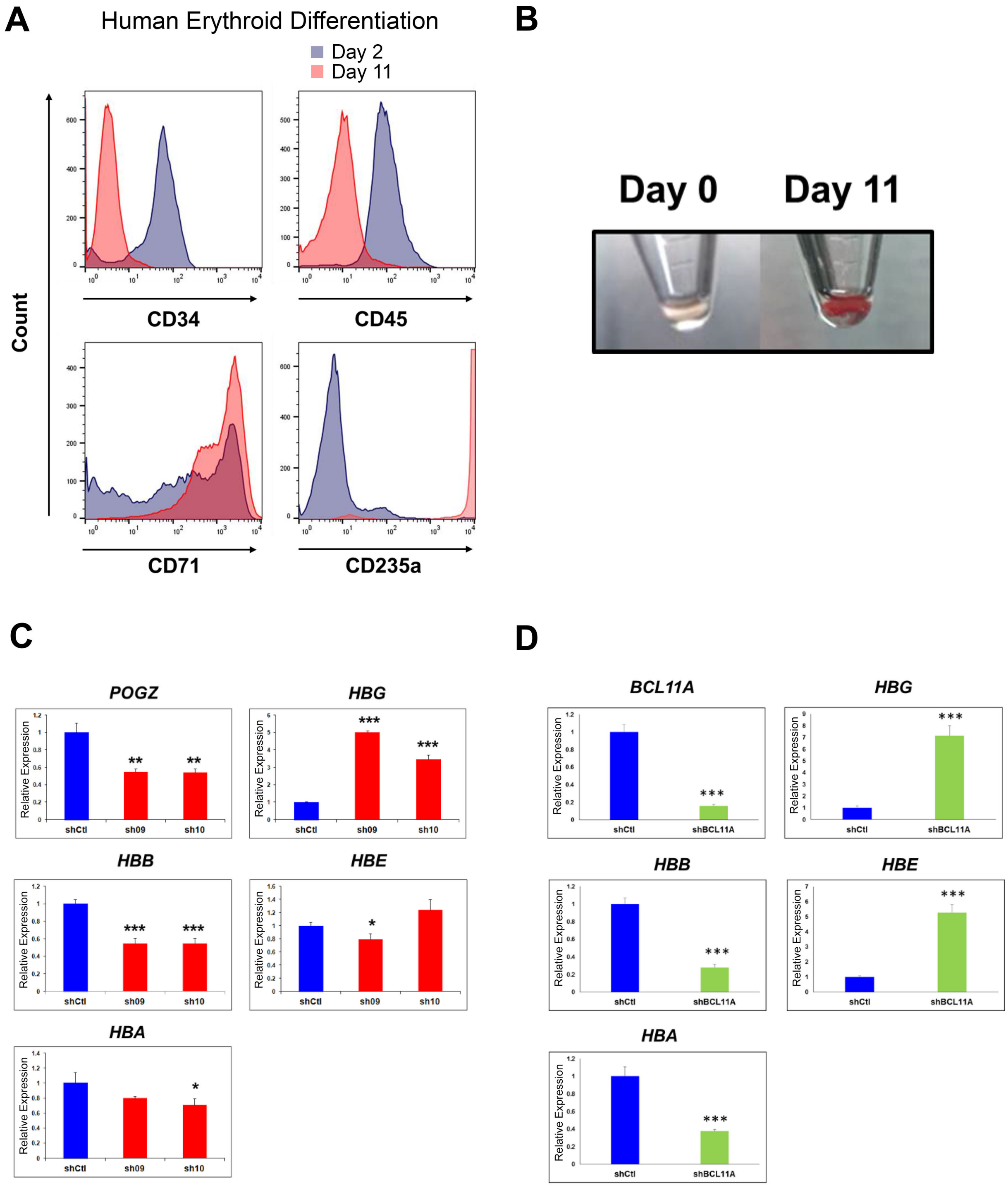
A
Human Erythroid Differentiation
Day 2
Day 11
Count
CD34
CD45
CD71
CD235a
B
Day 0
Day 11
C
POGZ
HBG
HBB
HBE
HBA
Relative Expression
shCtl
sh09
sh10
D
BCL11A
HBG
HBB
HBE
HBA
shCtl
shBCL11A

Figure S7

Figure S7. POGZ regulates fetal hemoglobin expression in normal human erythroid expansion and differentiation culture *in vitro*, Related to Figure 7. CD34+ cells were purified from mobilized peripheral blood cells, and then cultured in a modified 2 phase *in vitro* erythroid expansion and differentiation system. (**A**) Flow cytometry analysis of CD34, CD45, CD71 and CD235a expression on day 2 (blue histograms) and day 11 (red histograms) of erythroid cell cultures *in vitro*. (**B**) Photographs of undifferentiated control cell pellet at day 0 (left panel) and cell pellet of differentiated cells at day 11 demonstrating hemoglobinization of cells in culture (right panel). (**C**) Bar graphs showing expression of *POGZ*, *HBG1/2*, *HBB*, *HBE* and *HBA* following lentiviral mediated shRNA knockdown of *POGZ* using two POGZ shRNA constructs. Erythroblasts were harvested on day 10 post transduction, and expression analyzed by real time RT-qPCR. Gene expression was normalized to *ACTIN* expression. Experiments were performed in triplicates and data are presented as mean ± SD. * $P < 0.05$; ** $P < 0.01$; *** $P < 0.001$. (**D**) Bar graphs showing expression of *BCL11A*, *HBG1/2*, *HBB*, *HBE* and *HBA* following lentiviral mediated shRNA knockdown of *BCL11A*. Erythroblasts were harvested on day 10 post transduction, and expression analyzed by real time RT-qPCR. Gene expression was normalized to *ACTIN* expression. Experiments were performed in triplicates and data are presented as mean ± SD. *** $P < 0.001$.

Supplemental Experimental Procedures

Cell Cultures: KG1 cells were maintained in IMDM media (Life Technologies, Gaithersburg, MD, USA) containing 20% FBS and 1% Penicillin/Streptomycin (Life Technologies), and differentiated into myeloid cells by adding 20 ng/ml PMA (Sigma-Aldrich, St. Louis, MO, USA) and 20 ng/ml TNF-α (R&D Systems, Minneapolis, MN, USA). Murine erythroleukemia (MEL) cells were maintained in RPMI-1640 media (Life Technologies) containing 10% FBS and 1% Penicillin/Streptomycin (Life Technologies). Human CD34+ cells were obtained from peripheral blood stem/progenitor cells mobilized by granulocyte colony-stimulating factor (G-CSF) under studies (02-H-0160 and 08-H-0156) that were approved by the institutional review boards (IRB) of the National Heart, Lung, and Blood Institute (NHLBI) and the National Institute of Diabetes, Digestive, and Kidney diseases (NIDDK). All patients gave written informed consent for the sample donation and consent documents are maintained in the medical records. The consent documents were approved by the IRB prior to study initiation and were reviewed and updated yearly. The CD34+ cells were thawed and cultured in a 2 stage culture system as previously described (Migliaccio et al., 2002). Briefly, the cells were cultured in an expansion media consisting of IMDM with 1% Penicillin/Streptomycin, 20% FBS, 10 ng/ml Stem Cell Factor (SCF), 2.0 U/ml Erythropoietin (EPO), 1 ng/ml IL-3, 1 µM Dexamethasone and 1 µM Estradiol, for 5 days, with fresh media added as needed to keep the cells at 0.1-1x10^6 cells/ml. On day 6 cells were transferred to differentiation media consisting of IMDM, 1% Penicillin/Streptomycin,

20% FBS, 2.0 U/ml EPO, 10 ng/ml Insulin, 0.5 mg/ml Holo Transferrin and 2% BSA Fraction V. The differentiation media was changed every other day until cells were harvested.

Western Blot Analysis: Whole-cell lysates for western blot analysis were prepared from KG1 cells, MEL cells, wild type adult mouse tissues, Pogz+⁄+, Pogz+/- and Pogz−⁄− fetal brains, fetal livers and CD34 HSPC derived eryhtroblasts. The lysates were resolved on 4-12% Tris-Glycine pre-cast gels (Life Technologies), transferred to nitrocellulose membranes and probed with specific antibodies against Pogz (from Bethyl Laboratories, Montgomery, TX, USA), Bcl11a (Abcam, Cambridge, MA), fetal hemoglobin; HBG1/2 (Santa Cruz Biotechnology, Inc., Dallas, TX, USA) and Actin (Millipore, Billerica, MA, USA). Goat anti-mouse IgG-HRP or goat anti-rabbit IgG-HRP (Santa Cruz Biotechnology) were used as secondary antibodies, and ECL detection kit (GE Healthcare Biosciences, Pittsburgh, PA, USA) to develop blots.

Immunofluorescence: MEL cells were fixed with 2% paraformaldehyde on poly-L-lysine coated slides followed by permeabilization with 0.05% Triton X-100. The cells were blocked with 5% normal goat serum and 3% BSA for 30 minutes at room temperature. Cells were then labeled with primary antibodies to Pogz (1:50; IHC-00712, Bethyl Laboratories) and fibrillarin (1:200; sc-166021, Santa Cruz Biotechnology) overnight at 4°C. After washing with PBS, cells were incubated with Texas Red goat anti-rabbit (1:500; Invitrogen) and Alexa-Fluor 488 goat anti-mouse (1:500; Invitrogen) at room temperature for 1 hour. Cells were mounted with ProLong Gold anti-fade reagent with DAPI to stain for the nucleus and visualized by a LSM 710 scanning confocal microscope (Zeiss).

Mice: Mice were housed, fed and handled in accordance with the National Institutes of Health guidelines for animal care and use, and the Guide for the Care and Use of Laboratory Animals,

8th Edition. All mouse experiments were reviewed and approved by the Institutional Animal Care and Use Committee of the National Cancer Institute at Frederick, which is accredited by AAALAC International.

Targeted Disruption of the Pogz Gene: The conditional knockout vector for Pogz was constructed using the recombineering method as previously described (Liu et al., 2003). Briefly, using probes for the 5´and 3´ ends of the gene, three different 129/SV BAC clones (543F12, 60907, 529K9; all from Life Technologies) which contained the gene (or parts of the gene) were identified. BAC 60907 was found to contain the entire region of interest and was used for sequence retrieval. Primers used in the study were designed using MacVector (MacVector, Inc., Cary, NC, USA) and are listed in Table S2. All PCR reactions were performed using the ROCHE Expand High-Fidelity Kit (Roche Applied Science, Indianapolis, IN, USA). Exons 13-19, which encode the CENPB and DDE domains and a portion of the zinc finger region of the Pogz protein, were targeted. These were flanked by a loxP site at the 5′ end and a FRT-Neo-FRT-loxP cassette on the 3′ end. The Pogz targeting vector was linearized and introduced into CJ7 ES cells by a standard electroporation method. ES cell clones resistant for G418 and FIAU were selected and analyzed using Southern blot hybridization of EcoRI-digested genomic DNA. Two independently targeted ES cell clones were injected into C57BL/6 blastocysts to produce chimeras demonstrating germ-line transmission of the targeted gene. To delete the neo cassette from the line, Pogzneo/neo mice were crossed with β-actin-flp mice to produce Pogzf/+ mice. These were subsequently crossed to β-actin-cre mice to produce heterozygous Pogz+⁄− mice. To detect the full length Pogz coding sequence, cDNA from mouse fetal brain was amplified using the Advantage 2 PCR system (Clontech Laboratories, Inc. Mountain View, CA, USA) according to the manufacturer’s recommendations. Primer sequences are listed in Table S2. To

conditionally delete the Pogzf allele, an Mx1-cre+ transgene was introduced into Pogzf/f mice to generate Pogz+/+; Mx1-cre and Pogzf/f; Mx1-cre mice.

Microarray analysis: Microarray analysis was performed at the NCI Frederick Laboratory of Molecular Technology. Briefly, RNA was isolated from E14.5 Pogz+/+ and Pogz-/- fetal livers (3 of each genotype) using the Ambion RNAqueous-4PCR Kit (Life Technologies). RNA quality was analyzed using Agilent 2100 Bioanalyzer (Agilent Technologies Inc., Santa Clara, CA, USA). Approximately 100 ng RNA was reverse transcribed from each sample, labeled with biotin and hybridized to Affymetrix GeneChip Mouse Genome 430 2.0 microarrays (Affymetrix, Santa Clara, CA, USA). Data was analyzed using the GeneSpring software (Agilent Technologies Inc.). The cutoff value was set at ±1.5 fold and the false discovery rate at P=0.05. Of the 1232 transcripts with an Id, 1185 could be mapped and 1062 were analysis ready. Pathway analysis was performed using Ingenuity Pathway Analysis (IPA) (Ingenuity Systems, Inc., Redwood City, CA, USA). Microarray data was deposited to the Gene Expression Omnibus database (https://www.ncbi.nlm.nih.gov/geo/). Accession number GSE113503.

Flow Cytometry: For flow cytometric analysis of mouse cells, single-cell suspensions were prepared from E14.5-E16.5 Pogz+/+ or Pogz-/- FL cells, or BMC and PBC from animals transplanted with Pogz+/+ or Pogz-/- FL cells, or Pogz+/+; Mx1-cre and Pogzf/f; Mx1-cre BMCs. Where applicable, the cells were incubated with ACK lysis buffer (Life Technologies) to lyse red cells followed by a wash in PBS/2% BSA buffer. FL cells were resuspended in PBS/2% BSA and treated with Fc receptor blocking antibodies (anti-mouse CD16/32), and then incubated with the following conjugated monoclonal antibodies for lineage analysis: Isotype controls: rIgG2a (eBR2a), rIgG2b (eB149/10H5), CD71 (R17217), Ter119 (TER-119), anti-Gr-1 (RB6-8C5), anti-Mac-1 (M1/70), CD19 (1D3). For HSPC analysis, BMCs were incubated with

biotinylated antibodies against Lin markers (Mac-1, Gr-1, B220, Ter119, CD4, CD8), PE-conjugated c-Kit (2B8), APC-conjugated Sca-1 (E13-161.7), PE-Cy7-conjugated Flk2 (A2F10) FITC–conjugated CD34 (RAM34), PE-Cy5-conjugated IL-7r (A7R34), and streptavidin-eflour450. HSCs are Lin-negative (L), c-Kit+ (K) and Sca-1+ (S) (LSK) CD34-Flk2- cells, and common lymphoid progenitors (CLP) are LSK Flk2+IL7r+ cells. LK progenitors were isolated by replacing Flk2 and IL7r antibodies with PE-Cy7-conjugated FcγRII/III (2.4G2) including common myeloid progenitors (CMP) LK CD34+FcR-, granulocyte/macrophage progenitors (GMP) LK CD34+, FcR+, and megakaryocyte-erythrocyte progenitors (MEP) are LK CD34-FcR-. Megakaryocyte progenitors, (MP) progenitors are LK CD150+CD41+ cells, and preMegE are LK CD41-CD150+CD105- and were identified using PE-conjugated CD41 (MWReg30), APC-conjugated CD150 (mShad150), biotin-conjugated CD-105 (MJ7/18), and streptavidin-eflour450. Throughout the staining process, the cells were kept at 4°C, and the antibodies were used at a concentration of 0.2-0.5 μg/1 × 10^6 cells. For flow cytometric analysis of human CD34+ cells differentiated towards the erythroid lineage, cells were resuspended in PBS/2% BSA and treated with Fc receptor blocking antibodies (anti-human CD16/32), and then incubated with the following conjugated monoclonal antibodies for lineage analysis: FITC and APC-conjugated-mIgG1 (MOPC-21), APC-conjugated mIgG2a (G155-178), PE-conjugated mIgG2b (27-35), FITC-conjugated CD34 (581), APC-conjugated CD45 (HI30), APC-conjugated CD71 (M-A712), PE-conjugated CD235a (HIR2). All antibodies were purchased from BD Biosciences (San Jose, CA, USA) or eBiosciences (San Diego, CA, USA). The cells were analyzed by

FACS-CantoII or FACSCalibur (BD Biosciences) and data analyzed using FlowJo software (Tree Star Inc., Ashland, OR, USA).

Fetal Liver and Bone Marrow Cell Transplantation: Fetal livers were harvested from E14.5-E16.5 Pogz+/+ and Pogz-/- embryos and BMCs were isolated from adult Pogz+/+; Mx1-cre and Pogzf/f; Mx1-cre mice and transplanted as previously described (Gudmundsson et al., 2014; Gudmundsson et al., 2012). Briefly, FL cells were dissociated in PBS 4° C by mechanical disruption, and femurs were flushed using 5 ml syringe with a 29G × ½ needle and cells were passed through through 0.45 μm mesh filters (Millipore) to obtain single cell suspensions. The cells were resuspended in PBS and FL cells were transplanted at 0.25×10^6 cells/ 0.2 ml, and BMC at 1×10^6/ 0.2ml. The cell suspension was injected into the tail vein of lethally irradiated (10 Gy) recipient mice (B6.SJL-Ptprca Pep3b/BoyJ, CD45.1, Charles River Laboratories) to track donor reconstitution. Six weeks after transplantation of Pogzf/f; Mx1-Cre BMCs and controls, the mice were injected with 300μg of polyinosinic–polycytidylic acid solution (pIpC) dissolved in physiological water (NaCl 0.9%) (tlrl-pic, Invivogen or P1530, Sigma-Aldrich) 2 or 3 times every other day. The day of last injection was defined as day 0.

Lentiviral mediated shRNA knockdown: Mouse and human Pogz and Bcl11a shRNA target sequences were derived from The RNAi Consortium (TRC) portal (http://www.broadinstitute.org/rnai/public/) for oligo generation (sequences listed in Table S2). Oligos containing the target sequences were purchased, annealed and cloned into the pLKO.1 puro vector according to TRC protocols (http://www.broadinstitute.org/rnai/public/resources/protocols). Infectious lentivirus was generated by transfecting the shRNA constructs and packaging plasmids (PMD2G and pCMV8.74) into 293T/17 cells using the LipoD293 transfection agent (SignaGen, Rockville,

MD, USA). Virus containing supernatants were collected 48 hours post transfection. To calculate viral titers, NIH3T3 cells were transduced with serial dilutions of viral supernatants. Media containing puromycin (2 μg/ml) or blasticidin (10 μg/ml) was added 48 hours post transduction and resistant colonies counted 3 days later. For Pogz or Bcl11a knockdown, MEL cells or human CD34+ HSPC derived erythroblasts were transduced with shRNA lentivirus by spinoculation. Briefly, lentivirus and cells (4:1 ratio) were spun at 2000 x g for 90 minutes at 37°C. At 24-48 hours post transduction, puromycin (2 μg/ml) or blasticidin (10 μg/ml) was added to the cultures. MEL cells were harvested at 72-96 hours post transduction and CD34+ HSPC derived erythroblasts 9-12 days post transduction for real-time qRT-PCR and western blotting.

Retroviral transduction. MSCV-mPogz-IRES-eGFP retroviral construct was produced at the NCI-Frederick Protein Expression Laboratory and used to generate infectious viral particles in Ecotropic Plat-E packaging cells. Briefly, Plat-E cells were transiently transfected with a control retrovirus or MSCV-mPogz-IRES-eGFP retrovirus using Fugene 6 (Roche) and viral supernatant harvested 48 hours post-transfection. To calculate viral titers, NIH3T3 cells were transduced with serial dilutions of viral supernatants and GFP positive colonies counted 48 hours post-transduction. E16.5 Pogz-/- fetal liver cells were transduced in 1:4 ratio with control or MSCV-mPogz-IRES-eGFP retrovirus on plates coated with Retronectin (Takara Bio Inc., Mountain View, CA, USA) in DMEM media containing 15% FBS, 1% P/S, SCF (100 ng/ml), IL-3 (6 ng/ml), IL-6 (10 ng/ml) and Polybrene (1/1000). Two consecutive transductions were carried out with 24 hour interval and cells harvested for RNA isolation 48-60 hours after the second transduction. MEL cells were transduced with MSCV-mBcl11a-IRES-eGFP retrovirus following lentiviral mediated Pogz knockdown. A second transduction was performed 24 hours later and

puromycin added to the cultures (2 μg/ml). Cells were harvested at 96 hours post transduction for real-time qRT-PCR and western blotting.

Statistical Analysis: Statistical analysis was performed using Graphpad Prism (GraphPad Software, Inc.). Unpaired student's t-test was used to calculate statistical significance. Results were considered significant if $P < 0.05$. Results are presented as mean ± standard deviation.

Chromatin immunoprecipitation (ChIP) assay: ChIP assay was performed on chromatin isolated from MEL cells using the ChIP-IT Express Enzymatic kit according to the manufacturers recommendations (Active Motif, Carlsbad, CA, USA). Briefly, 1.5×10^7 MEL cells were crosslinked in media containing 1% formaldehyde and nuclei extracted by incubating the cells in lysis buffer for 30 min on ice followed by dounce homogenization and incubation in enzymatic digestion for 10 min to shear the chromatin. Sheared chromatin was immunoprecipitated overnight at 4°C in a solution containing protein-G magnetic beads and anti-Pogz or rabbit IgG (both from Bethyl). Immunoprecipitated chromatin was washed, eluted from the beads and the crosslinking reversed. Purified DNA was used for qPCR with the following primers for the *Bcl11a* enhancer region: +58 ChIP-F: 5'- AAA GGT GTT GGG TTC TGA GG-3'; +58 ChIP-R: 5'- ATC AGC AGC GAG CTC TCA TA-3'. The following primers were used for the *Bcl11a* promoter region: mBcl11a (-3496)-F: 5'-AAG CCA TTT CTG GAG AGG TAA A-3'; mBcl11a (-3496)-R: 5'-GTA TTG TGG AGC TGG GTG AA-3'; mBcl11a (-972)-F: 5'-CTT CTC GGT CTA TGT ATT CCA ATC T-3'; mBcl11a (-972)-R: 5'-GAT CTG AGC GAC

CCT ACA AAC-3’. A validated mouse negative control primer set from Active Motif (negative primer set 2) was used as a negative control.

Reverse Phase - High Performance Liquid Chromatography (HPLC): Hemoglobin quantity (HBB, HBE, HBG1/2) in erythroblast cultures was determined by HPLC analysis using the Agilent 1100 HPLC series (Agilent Technologies, Santa Clara, CA) according to the manufacturers recommendations.
